# Potential of *Variovorax paradoxus* isolate BFB1_13 for bioremediation of BTEX contaminated sites

**DOI:** 10.1186/s13568-021-01289-3

**Published:** 2021-09-06

**Authors:** Tibor Benedek, Flóra Szentgyörgyi, Veronika Gergócs, Ofir Menashe, Perla Abigail Figueroa Gonzalez, Alexander J. Probst, Balázs Kriszt, András Táncsics

**Affiliations:** 1 Department of Molecular Ecology, Institute of Aquaculture and Environmental Safety, Hungarian University of Agriculture and Life Sciences, Gödöllő, Páter K. u. 1, 2100 Hungary; 2 Institute for Soil Sciences, Budapest, Herman Ottó út 15, 1022 Hungary; 3 Water Industry Engineering Department, Achi Racov School of Engineering, Kinneret Academic College on the Sea of Galilee, 15132 D.N. Emek Ha’Yarden, Israel; 4 BioCastle Water Technologies Ltd, Tzemah, Israel; 5 Group for Aquatic Microbial Ecology, Environmental Microbiology and Biotechnology, Faculty of Chemistry, University of Duisburg-Essen, Universitätsstr. 5, 45141 Essen, Germany; 6 Department of Environmental Safety, Institute of Aquaculture and Environmental Safety, Hungarian University of Agriculture and Life Sciences, Gödöllő, Páter K. u. 1, 2100 Hungary

**Keywords:** *Variovorax paradoxus*, BTEX, WGS, SBP encapsulation, Biobarrier

## Abstract

Here, we report and discuss the applicability of *Variovorax paradoxus* strain BFB1_13 in the bioremediation of BTEX contaminated sites. Strain BFB1_13 was capable of degrading all the six BTEX-compounds under both aerobic (O_2_ conc. 8 mg l^−1^) and micro-aerobic/oxygen-limited (O_2_ conc. 0.5 mg l^−1^) conditions using either individual (8 mg‧l^−1^) or a mixture of compounds (~ 1.3 mg‧l^−1^ of each BTEX compound). The BTEX biodegradation capability of SBP-encapsulated cultures (SBP—Small Bioreactor Platform) was also assessed. The fastest degradation rate was observed in the case of aerobic benzene biodegradation (8 mg l^−1^ per 90 h). Complete biodegradation of other BTEX occurred after at least 168 h of incubation, irrespective of the oxygenation and encapsulation. No statistically significant difference was observed between aerobic and microaerobic BTEX biodegradation. Genes involved in BTEX biodegradation were annotated and degradation pathways were predicted based on whole-genome shotgun sequencing and metabolic analysis. We conclude that *V. paradoxus* strain BFB1_13 could be used for the development of reactive biobarriers for the containment and in situ decontamination of BTEX contaminated groundwater plumes. Our results suggest that *V. paradoxus* strain BFB1_13—alone or in co-culture with other BTEX degrading bacterial isolates—can be a new and efficient commercial bioremediation agent for BTEX contaminated sites.

**Supplementary Information:**

The online version contains supplementary material available at 10.1186/s13568-021-01289-3.

## Key points

- First report on the BTEX biodegradation capacity of *V. paradoxus*
- No significant difference between aerobic and micro-aerobic BTEX biodegradation
- Strain BFB1_13 can be applied for in situ bioremediation of BTEX contaminated sites.

## Introduction

BTEX (benzene, toluene, ethylbenzene, *o*-, *m*- and *p*-xylene) compounds are of great concern as they are common water resource and potable water contaminants posing risk to the human health (Li et al. 2017). Their presence in groundwater is especially worrisome in vulnerable regions like Africa and South-East Asia, where the population is highly dependent on drinking water from wells and boreholes (Fayemiwo et al. 2017).

BTEX are treated as priority pollutants according to U.S. EPA. They are included in the Hazardous Air Pollutants List (rank 78) in the CERLA List from the 275 substances identified as significant threats to human health (Rahul and Balomajumder 2013). Benzene and ethylbenzene, have adverse health effects including cancer induction, and neurological effects like weakness, fatigue, loss of appetite, confusion, and nausea (Smith et al. 2016; WHO 2010). Toluene, *o*-, *m*- and *p*-xylene have severe acute effects of exposure such as neurotoxicity and reproductive problems (Wilbur and Bosch 2004).

In spite of their adverse health effects, BTEX are among the most abundantly used chemicals worldwide in petroleum, solvent, paint, adhesives, rubber and pesticides industries (Abumaizar et al. 1998; Atlas and Philp 2005; Fayemiwo et al. 2017). The main sources of BTEX pollution include petroleum industry, leakage of crude oil and petroleum derivatives, coal and biomass burning, paint manufacturing and application (Kelley et al. 1997; Baltrenas et al. 2011; Mitra and Roy 2011; Zhang et al. 2020; Datta et al. 2013). According to a recent study, leachates of cigarette butts can also be serious sources of contamination of water bodies with BTEX compounds (Dobaradaran et al. 2021). In the urban atmosphere they are the most abundant volatile organic compounds (VOCs) (Duan and Li 2017; Dehghani et al. 2018). In summary, BTEX pollution threatens all the three spheres, the pedosphere, hydrosphere and the atmosphere (Andreoni and Gianfreda 2007; Benedek et al. 2016; Farkas et al. 2017).

Based on the aforementioned, it is evident that the minimization of BTEX emissions and remediation of already contaminated matrices play a critical role in human and environmental health. Over the years a series of studies, including advanced oxidation technologies, photocatalysis, sonolysis, radiolysis etc., have been conducted to improve BTEX removal efficiencies (Yerushalmi et al. 1999; Mascolo et al. 2008; Braeutigam et al. 2009; Saponaro et al. 2009; Laokiat et al. 2012; Lee et al 2013; Caetano et al. 2016; Al-Sabahi et al. 2017; Lin et al. 2017; Dhivakar and Vel rajan 2018; Mohan et al. 2020, Vaezihir et al. 2020). However, still the most popular technique is bioremediation, especially when it is applied in situ, because it is the most cost-effective means of removing many contaminants including BTEX (Cunningham et al. 2001; Rifai 2005; Singh et al. 2017; Lee et al. 2019). Although it has been utilized for decades, bioremediation still holds innovative solutions. Amongst the most innovative approaches, the Small Bioreactor Platform technology (SBP) deserves special attention as an emerging biotechnological tool. In an SBP capsule the biotechnologically-relevant microorganisms can exert their beneficial metabolic properties in a confined and protected environment. In the absence of competition with the endogenous microbial community of the contaminated site, the survival rate and thus the efficiency of introduced bacteria within an SBP capsule can be significantly higher (Menashe and Kurzbaum 2014; Menashe et al. 2020a, b).

All the above-mentioned facts indicate the importance of discovering new microbial capabilities for BTEX biodegradation. In this study, we aimed at determining the BTEX biodegradation potential of a recently isolated *Comamonadaceae* bacterium, *Variovorax paradoxus* strain BFB1_13. Although according to the review of Satola et al. (2013) *V. paradoxus* affiliated isolates are capable of degrading a wide variety of recalcitrant organic pollutants (e.g., PAHs, PCBs, dinitrotoluene, and trichloroethylene), there exists—to the best of our knowledge—no information in the literature regarding BTEX biodegradation ability of bacterial isolates related to *V. paradoxus*. Moreover, to date there is a lack of information regarding the simultaneous biological degradation of the full range of BTEX by pure *Variovorax* isolates, although *Variovorax* spp. are often detected in BTEX contaminated environments (Hendrickx et al. 2006; Benedek et al. 2016, 2018; Posman et al. 2017).

In this study, we assessed the applicability of *V. paradoxus* strain BFB1_13 in environmental remediation using a polyphasic approach based on microcosm experiments and whole-genome shotgun sequencing (WGS). Based on WGS, gene annotations and metabolic analyses, we attempted to elucidate BTEX degradation pathways of strain BFB1_13 for each compound. By using planktonic and SBP encapsulated cultures of strain BFB1_13, we investigated aerobic and micro-aerobic/oxygen-limited biodegradation of BTEX compounds. The results increase understanding of BTEX degradation of the genus *Variovorax* and, in the long-term, could contribute to the restoration of BTEX contaminated sites through bioaugmentation with the studied isolate.

## Materials and methods

### Brief characterization of *Variovorax paradoxus* strain BFB1_13

*Variovorax paradoxus* strain BFB1_13 was previously isolated from a bacterial biofilm community selectively enriched on a mixture of BTEX compounds as sole carbon and energy source (50 mg l^−1^, ratio of individual BTEX compounds was 1:1) in an aerobic, vitamins and trace elements amended mineral salts medium. Originally, the source biofilm had developed in a BTEX contaminated hypoxic groundwater on the surface of a stainless-steel submersible pump belonging to a Pump and Treat system (Benedek et al. 2016, 2018).

Strain BFB1_13 when grown on R2A agar plate forms large, yellowish colonies (more accentuated color in the middle), with irregular shape and raised elevation. The colonies with irregular margins have high ability of swarming. Strain BFB1_13 possesses catechol 2, 3-dioxygenase encoding gene (*C23O*) belonging to subfamily I.2.C, which is involved in the oxygen-limited degradation of simple aromatic hydrocarbons (Kukor and Olsen 1996). The *16S rRNA* and *C23O* gene sequences of the strain can be accessed under the accession numbers MG897130 and MG926652, respectively (Benedek et al. 2018). *V. paradoxus* strain BFB1_13 is deposited in the National Collection of Agricultural and Industrial Microorganisms (NCAIM, Budapest, Hungary) under the accession number NCAIM B.02666.

### Optical density/colony forming units (CFU) determination

At different stages of the study (optimization of cultivation conditions and biodegradation experiments) bacterial suspensions of different optical densities measured at 600 nm (OD_600nm_) were used (0.5 and 1). Therefore, prior to subsequent investigations, in order to know the exact bacterial cell numbers used for a given experiment, OD_600nm_/colony forming unit (CFU) determinations were conducted.

From actively growing cultures of *V. paradoxus* BFB1_13, cultivated on R2A agar, bacterial suspensions of OD_600nm_ 0.5 and 1 were prepared in 0.9% saline solution. Subsequently, the obtained bacterial suspensions were serially diluted up to 10^8^. Dilutions of 10^4^ up to 10^8^ were spread (100 µl) onto R2A agar plates in three replicates (for the composition of the used culture medium please see Additional file 1: Table S1). After 48 h of incubation the grown colonies were enumerated.

### Optimization of cultivation conditions, tolerance tests

In order to find the most appropriate cultivation conditions and at the same time to determine the tolerance of the investigated bacterium to temperature, pH and NaCl, three different culture media (nutrient-broth, R2A and TSA growth media), six different temperatures (4, 10, 15, 25, 30, and 37 °C), 10 different pH values (2, 4, 5, 6, 7, 8, 9, 10, 11 and 12, the growth media’s pH was adjusted with 37% HCl or 10% KOH) and 12 different NaCl concentrations (0, 1, 2, 3, 4, 5, 6, 7, 8, 9, 10 and 12%) were tested. The composition of the used growth media is presented in Additional file 1: Table S1.

Each time, after revitalization of the bacterium from − 80 °C cryopreserved stock bacterial suspensions, actively growing cultures of strain BFB1_13 on R2A agar plates were used. Prior to each test, bacterial suspensions of OD_600nm_ = 0.5 were prepared in 0.9% saline solutions. Test solutions (50 ml) were inoculated with 100 µl of bacterial suspension and shaken at 145 rpm. Schott borosilicate glass bottles (100 ml) were used. The turbidity of the cultures was determined spectrophotometrically at 600 nm (OD_600nm_), measured in every 2 h. The temperature tolerance tests were conducted together with the selection of the most appropriate culture medium at pH 7. For pH and NaCl tolerance tests, nutrient-broth was used. The shaking cultures were incubated at 30 °C.

### Investigation of biofilm forming ability of the strain by using CDC reactor

Previously, the biofilm-producing ability of *V. paradoxus* strain BFB1_13 was assessed by using cell culture chimney 96-well microplates and the crystal-violet assay. Based on previous results, isolate BFB1_13 proved to be weakly adherent to the polystyrene wall of the microplate (Benedek et al. 2018).

In this study the biofilm producing capability of the strain was further assessed in batch cultures by using a CDC-biofilm reactor (Goeres et al. 2005). Thirteen different biofilm forming surfaces (coupons, 12.7 mm diameter) were used: polished stainless steel, hydroxyapatite, natural rubber, viton rubber, polycarbonate, copper, stainless steel, ductile iron, polypropylene, polyvinyl chloride, polytetrafluoroethylene, titanium and glass (BioSurface Technologies Corporation, Montana, USA). 350 ml of nutrient-broth in the CDC reactor was sterilized in autoclave (121 °C 1 atm, 20 min) together with the biofilm coupons. After sterilization, the growth medium was inoculated with 1 ml of bacterial suspension (OD_600nm_ = 1). Inoculated CDC reactors were incubated at 28 °C at 120 rpm for 6 days under batch conditions. After incubation, the biofilm forming ability of the strain on a given surface was assessed by using crystal-violet assay adapted from Kumari et al. (2013). Briefly, coupons were removed aseptically from the coupon holder rods and placed into 50 ml Falcon tubes, and were washed thrice with 5 ml phosphate buffer saline (pH 7.2) in order to remove planktonic cells. Coupon-attached cells were fixed with methanol (99%, 5 ml) for 15 min. The methanol was then discarded and the attached cells were stained with crystal-violet for 20 min (0.5%, 5 ml). Coupons were again decanted and washed thrice with distilled water (20 ml). The stained cells adherent to the coupons were resolubilized with 33% glacial acetic acid (5 ml). The absorbance of the obtained solutions was determined at 550 nm using a spectrophotometer (Implen GmbH, Germany). Biofilm production of the strain BFB1_13 was tested in three independent replicates for each coupon. Coupons without bacteria, which were those not placed in the growth medium, were used as negative-controls and were subjected to the same treatment as the positive, bacterial biofilm-containing coupons.

### Assessing the BTEX biodegradation ability of strain BFB1_13 by using planktonic cultures

As a first step, the aromatic hydrocarbon degradation potential of the isolate was tested aerobically in microcosm experiments by using as sole carbon and energy source one BTEX compound at a time or a mixture of BTEX compounds (microcosm experiment No 1). Afterwards, as second step in studying this strain, the degradation potential of the isolate for BTEX mixture was reassessed under aerobic (O_2_ conc. 8 mg l^−1^) and oxygen-limited conditions (O_2_ conc. 0.5 mg l^−1^, microcosm experiment No 2). Due to the slow BTEX degradation observed, the aerobic and oxygen-limited biodegradation experiments were repeated by using more biomass for inoculation (1 ml of inoculum instead of 100 μl in 50 ml of test media) containing younger bacterial cells (24 instead of 72 h old culture; microcosm experiment No 3).

During the microcosm experiments, in hermetically closed, crimp sealed serum bottles, 50 ml of Bushnell-Haas (BH) medium contained either individual (final concentration of 8 mg l^−1^) or a mixture of BTEX compounds (total BTEX concentration was 8 mg‧l^−1^; ~ 1.3 mg‧l^−1^ of each BTEX compound). The composition of BH medium was the following: CaCl_2_ · 2H_2_O 0.002 g, MgSO_4_ · 7H_2_O 0.02 g, NH_4_NO_3_ 1 g, KH_2_PO_4_ 1 g, K_2_HPO_4_ 1 g, FeCl_3_ ∙ 6H_2_O 0.005 g, H_2_O 1 l, with pH 7. Shaking test solutions were inoculated with bacterial suspensions (100 µl or 1 ml, OD_600nm_ = 1), previously grown for 24 or 72 h, obtained in normal saline solution. Microcosms were incubated at 28 °C while shaking at 145 rpm. Non-inoculated microcosms were also incubated to be used as abiotic controls. All the microcosm experiments were conducted in triplicates. Oxygen-limited conditions were set as described in our previous study. Briefly, oxygen-limited microcosms were sparged aseptically with N_2_:CO_2_ gas and the desired oxygen concentration was set by sterile air injection into the bottles. Oxygen concentration was measured from the liquid phase by using Fibox 3 trace v3 fiber optic oxygen meter with PSt3 sensor spots (Benedek et al. 2018).

BTEX concentration during the experiments, was determined from the headspace using an SPME polydimethylsiloxane fiber assembly (Supelco) for sampling and a Trace 1300 gas chromatograph coupled to ISQ Single Quadrupole mass spectrometer (ThermoFisher Scientific) for analysis. During the analysis, injector and detector temperatures were maintained at 200 °C and 250 °C, respectively. The oven temperature program was set to 40 °C for 3 min then ramped at a rate of 20 °C min^−1^ to 190 °C and finally held for 1 min. Helium was used as carrier gas at a flow rate of 1.2 ml min^−1^. SLB™-5 ms fused silica capillary column was used for separation (30 m × 0.25 mm × 0.25 μm, Sigma-Aldrich, Supelco). The mass spectrometer was operated at full scan mode.

### Determination of BTEX biodegradation capability of the SBP encapsulated culture

BTEX biodegradation ability of SBP encapsulated BFB1_13 cells was determined under aerobic conditions (microcosm experiment No 4). Again, OD_600nm_ = 1 bacterial suspension in saline-R2A (2%) solution was prepared by using actively growing cultures (72 h). 1 ml of bacterial suspension was added to SBP capsules under sterile conditions by using sterile syringe and needle following the instructions of the manufacturer (Research Kit, Catalog number AC-20, BioCastle Ltd., Israel; https://www.youtube.com/watch?v=yEMjx2FZT5Y). After inoculum injection the needle hole was sealed with the provided polymer (cellulose acetate solution) by creating three layers membrane. After the sterilization of the outer surface (immersion of capsules in absolute ethanol for 5 s), the inoculated SBP capsules were transferred to 50 ml crimp sealed serum bottles containing 50 ml of BH medium and BTEX mixture (total 8 mg l^−1^; 1:1, v/v, the concentration of individual BTEX compounds was ~ 1.3 mg l^−1^). One SBP capsule was added into each bottle. Microcosms were incubated at room temperature (25 °C) shaking at 145 rpm. Negative controls, containing SBP capsules without bacterial culture, were also incubated. The concentration of BTEX in the headspace of the bottles was determined as described in "Assessing the BTEX biodegradation ability of strain BFB1_13 by using planktonic cultures" section. The experiment was conducted in four replicates.

### Statistical analyses

To reveal the temporal change of BTEX degradation process in the aerobic and micro-aerobic microcosms, the temporal data of concentration reductions were fitted with cubic spline functions (microcosm experiment No 2; Additional file 1: Table S2). Based on this, three types of functions could be fitted to the degradation processes of the six compounds. Logistic and saturation curves, as well as linear models were fitted to the data sets. All the analyses were performed in R program (R Core Team 2019). Spline fittings and general linear models were carried out with compound symmetry correlation in gls function; and four-parameter logistic; and three parameter saturation fittings were carried out with gnls function (nlme package: Pinheiro et al. 2019). The fitted curves were compared between the aerobic and micro-aerobic systems (microcosm experiment No 2). Curve types were selected according to regression diagnostics performed with plot and qqnorm functions, and with the lowest SSE values.

In the case of the repeated aerobic/micro-aerobic BTEX degrading microcosm experiment (microcosm experiment No 3), fewer measurements were conducted than in the first one, and thus, no curves could be fitted on the second data set. Therefore, concentration reduction values at the 168th hour were compared between the first and the second experiment and between aerobic and micro-aerobic systems (microcosm experiment No 2 vs. 3) with Kruskal–Wallis test in kruskal.test function (R Core Team 2019).

### Whole-genome sequencing and analysis

Genomic DNA of strain BFB1_13 was extracted using the DNeasy® UltraClean® Microbial Kit (Qiagen, Hilden, Germany) and sequenced as described earlier in Borsodi et al. (2019). Briefly, Nextera Mate Pair Sample Preparation Kit (Illumina, U.S.A) was used to generate mate-paired libraries according to the manufacturer's protocol for gel-plus version with slight modifications. 13 µl of Mate-Paired Tagment Enzyme was used to produce a robust smear within the 7–11 kbp region. The 7–11 kbp DNA fraction was excised from the gel using the Zymoclean Large Fragment DNA Recovery kit (Zymo Research, U.S.A) and the circularized DNA was sheared using Covaris S2. All quality measurements were performed on a TapeStation 2200 instrument (Agilent, U.S.A). Final libraries were quantified using Qubit (ThermoFisher, U.S.A) and sequenced on an Illumina MiSeq instrument using MiSeq Reagent Kit v2 (500 cycles) sequencing chemistry. De novo assembly and scaffolding were performed with CLC Genomics Workbench Tool v11 (Qiagen, Germany). The mate-paired reads were assembled into 617 contigs.

Annotation of the genome was performed by the Microbial Genome Annotation & Analysis Platform MicroScope (MaGe, Vallenet al. 2020). Additionally, putative functions of genes associated in the metabolism of xenobiotics were identified and bioinformatically analyzed by using MaGe in conjunction with the UniProt database (http://www.uniprot.org/; The UniProt Consortium 2019) and BLAST searches.

To ascertain the precise taxonomic position of the strain, the average nucleotide identity (ANI) and in silico DNA-DNA hybridization values between strain BFB1_13 and closest relatives were calculated using Ez-Biocloud (https://www.ezbiocloud.net/tools/ani; Yoon et al. 2017) and the server-based genome-to-genome distance calculator version 2.1 (https://ggdc.dsmz.de/ggdc.php#; Meier-Kolthoff et al. 2013), respectively.

## Results

### Optical density versus bacterial cell numbers

Results of the OD_600nm_/CFU determination indicated that bacterial suspensions of isolate *V. paradoxus* BFB1_13 adjusted to OD_600nm_ 0.5 and 1 contain 2.3 · 10^8^ and 5.5 · 10^8^ CFU ml^−1^, respectively.

### Optimal cultivation conditions and the results of tolerance tests

Amongst the tested growth media, nutrient-broth proved to be the most suitable for the cultivation of the bacterium. The highest OD_600nm_ values were obtained in nutrient-broth incubated at 25 °C and 30 °C; OD_600nm_ values were 1.7 (after 53 h of incubation) and 1.8 (51 h of incubation), respectively. Although in R2A, incubated at 30 °C, a faster growth rate was observed, the maximum OD_600nm_ reached after 51 h of incubation was only 1.4. In R2A medium the cells entered the stationary phase early, after 36 h of incubation (Additional file 1: Fig. S1). Irrespective of the incubation temperature strain BFB1_13 showed a delayed growth in TSA medium. At 37 °C the bacterium did not show remarkable growth in any of the tested media (Additional file 1).

Based on these results, and to further determine the tolerance of the isolate at lower temperatures, as well as different pH levels and NaCl concentrations, the bacterium was grown in nutrient-broth.

Strain BFB1_13 was also able to grow at lower temperatures as determined at 4, 10 and 15 °C, although at a much slower rate. At lower temperatures a delayed growth was recorded. After 48 h of incubation growth was recorded only in the case of the growth medium incubated at 15 °C (OD_600nm_ 0.13). After 96 h of incubation the bacterium showed growth also at 4 and 10 °C, the recorded OD_600nm_ values were 0.015 and 0.163, respectively. At this time point the OD_600nm_ of the bacterial suspension incubated at 15 °C was quite high, 1.03. After 10 days of incubation the recorded OD_600nm_ values were 0.3 and 0.48 at 4 and 10 °C, respectively.

*Variovorax paradoxus* strain BFB1_13 was able to grow in nutrient-broth with pH values between 5 and 9 and NaCl concentrations of 0–2% (Additional file 1: Fig. S2). Outside of the mentioned pH and NaCl levels no growth of the bacterium was recorded.

### Biofilm-producing potential of strain BFB1_13

**Fig. 1:**
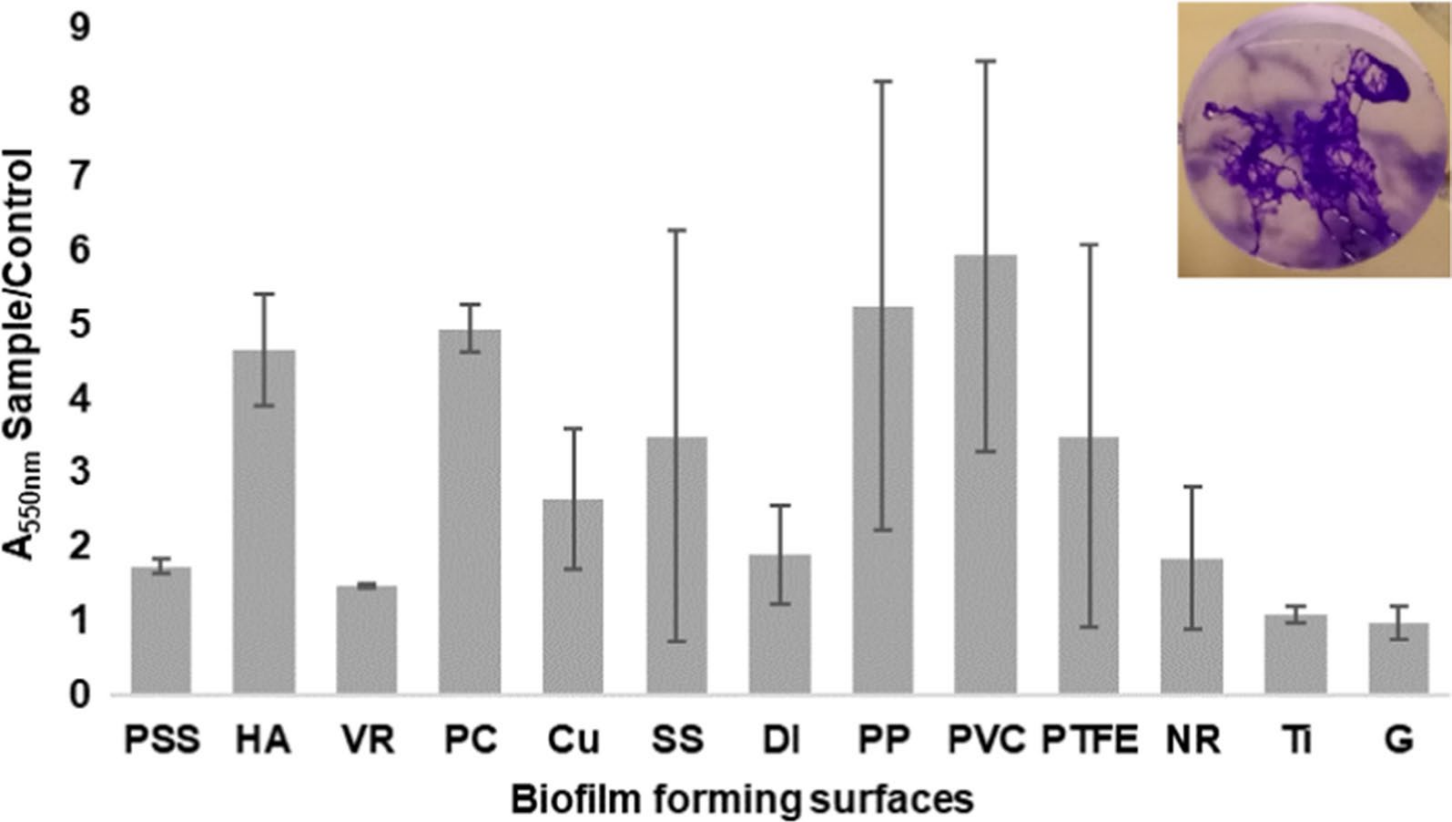
Biofilm forming ability of *V. paradoxus* strain BFB1_13 on polished stainless steel (PSS), hydroxyapatite (HA), viton rubber (VR), polycarbonate (PC), copper (Cu), stainless steel (SS), ductile iron (DI), polypropylene (PP), polyvinyl chloride (PVC), polytetrafluoroethylene (PTFE), natural rubber (NR), titanium (Ti) and glass (G). The ratio of absorbance values obtained at 550 nm (A_550nm_) of biofilm containing coupons (Sample) and abiotic control coupons (Control) is shown. A crystal-violet stained biofilm of strain BFB1_13, developed on polycarbonate surface is shown in the top right-hand corner. The average of three replicates is shown, error bars represent standard deviations (STD)

Strain BFB1_13 produced prolific biofilms on hydroxyapatite, polycarbonate, polypropylene and polyvinyl chloride. The absorbance values (550 nm) of the crystal-violet solutions originating from the treatment of biofilm containing coupons were at least 4 times higher than that of abiotic control coupons in at least two replicates. Notable biofilm production was observed also in the case of copper (threefold difference as compared to the control coupon), stainless steel (7.5-fold), polytetrafluoroethylene (sevenfold) and natural rubber (threefold), although the deviation between the replicates was high (Fig. 1). No remarkable biofilm formation occurred on polished stainless steel, viton rubber, ductile iron, titanium and glass surfaces (Fig. 1).

### BTEX biodegradation potential of strain BFB1_13

#### BTEX biodegradation of planktonic cultures

**Fig. 2:**
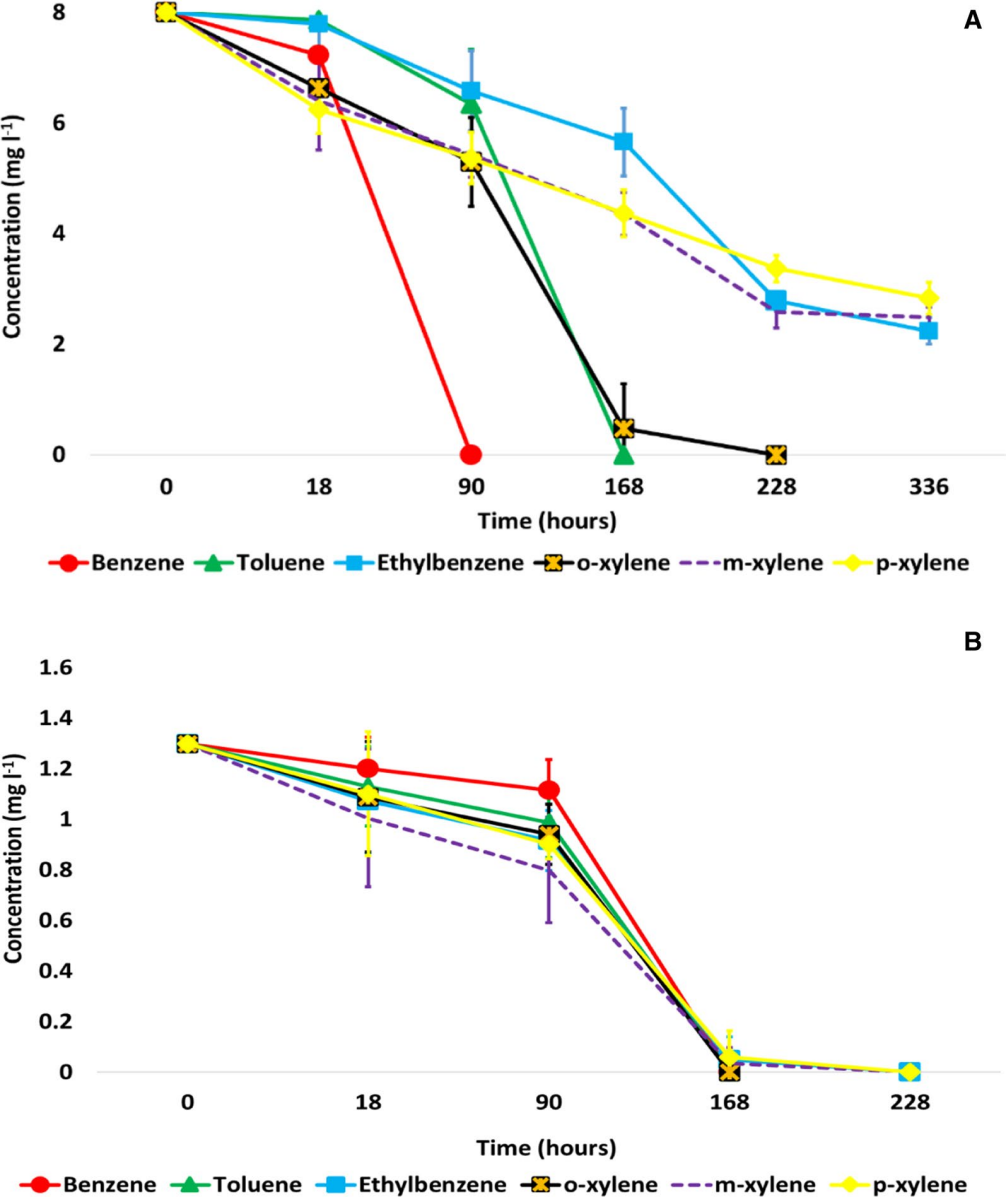
Individual (**A**) and BTEX mixture (**B**) degradation ability of *V. paradoxus* strain BFB1_13 under aerobic conditions

As it was expected from previous results (Benedek et al. 2018), strain BFB1_13 was able to degrade BTEX compounds. However, different BTEX biodegradation rates were observed when using individual or a mixture of BTEX compounds. In individual BTEX biodegradation experiments benzene proved to be the most susceptible to bacterial biodegradation, followed by toluene and *o*-xylene. Complete degradation of these compounds was recorded after 90, 168 and 228 h of incubation, respectively. However, in this setting no complete biodegradation of ethylbenzene, *m*-, and *p*-xylene occurred. After 14 days of incubation on average only 71.3% of ethylbenzene, 61.1% of *m*-xylene and 54.8% of *p*-xylene had been eliminated (mean values of three replicates, Fig. 2A). These reduction values were slightly higher than the abiotic loss of BTEX from microcosms, which was 36.5% for ethylbenzene, 41.4% for *m*- and *p*-xylene.

On the other hand, complete biodegradation of all BTEX was observed in mixture amended microcosms. When a mixture of BTEX was added to the bottles, complete benzene, toluene and *o*-xylene degradation occurred after 168 h, and complete ethylbenzene, *m*- and *p*-xylene biodegradation occurred after 228 h of incubation (Fig. 2B).

#### Aerobic and micro-aerobic BTEX mixture degradation of *V. paradoxus* strain BFB1_13

**Fig. 3:**
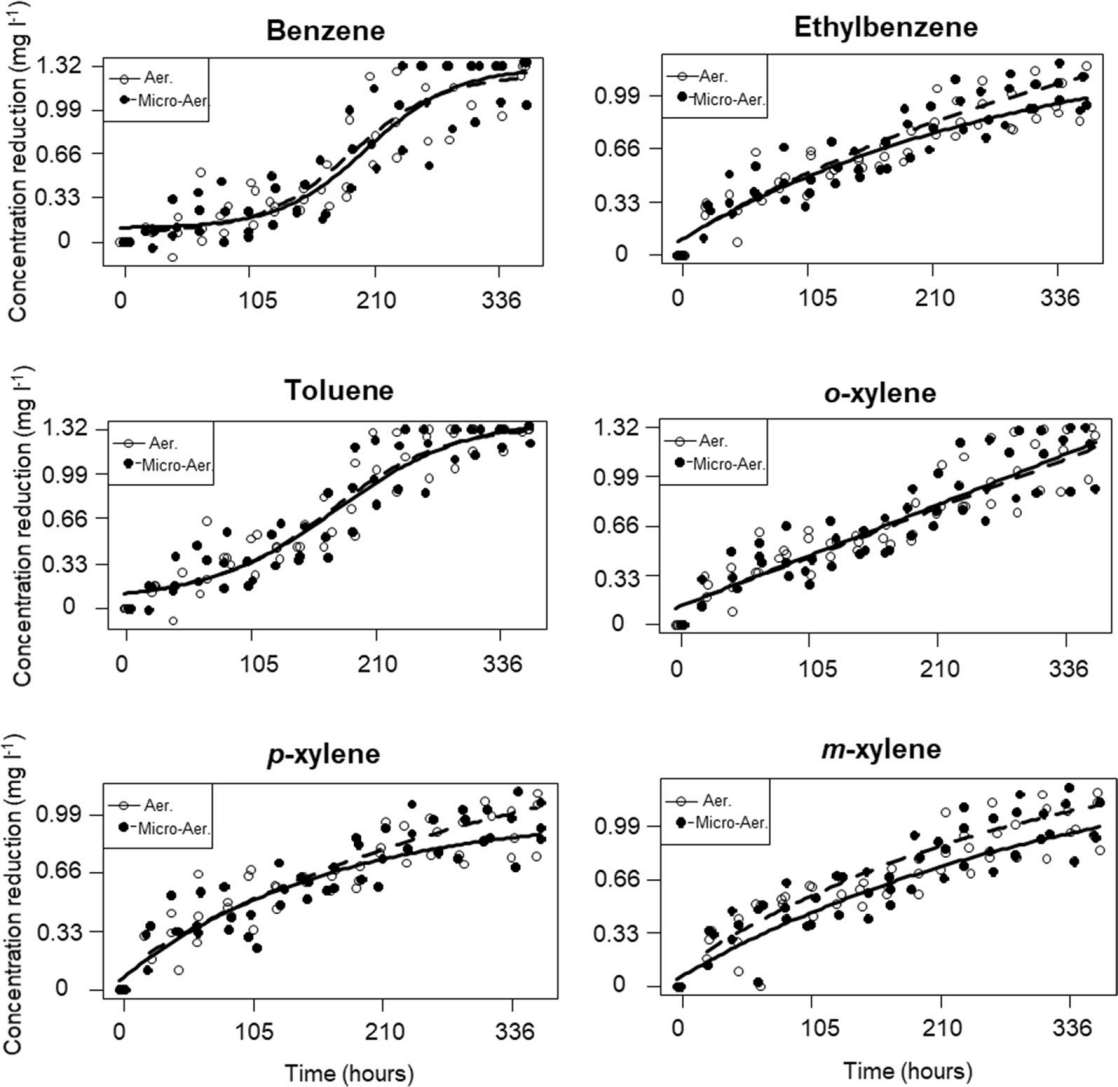
Fitted models of the degradation processes of the six BTEX compounds in the aerobic and micro-aerobic systems. Parameters of the models are displayed in Additional file 1: Table S2

According to the microcosm experiment No 2, biodegradation of the six compounds could be modelled with three different function models. Data of benzene and toluene could be described as logistic curves; ethylbenzene, *p*- and *m*-xylene could be modelled as saturation curve and *o*-xylene was fitted with linear regression model (Fig. 3). However, the differences were small between saturation curves and the linear model.

**Fig. 4:**
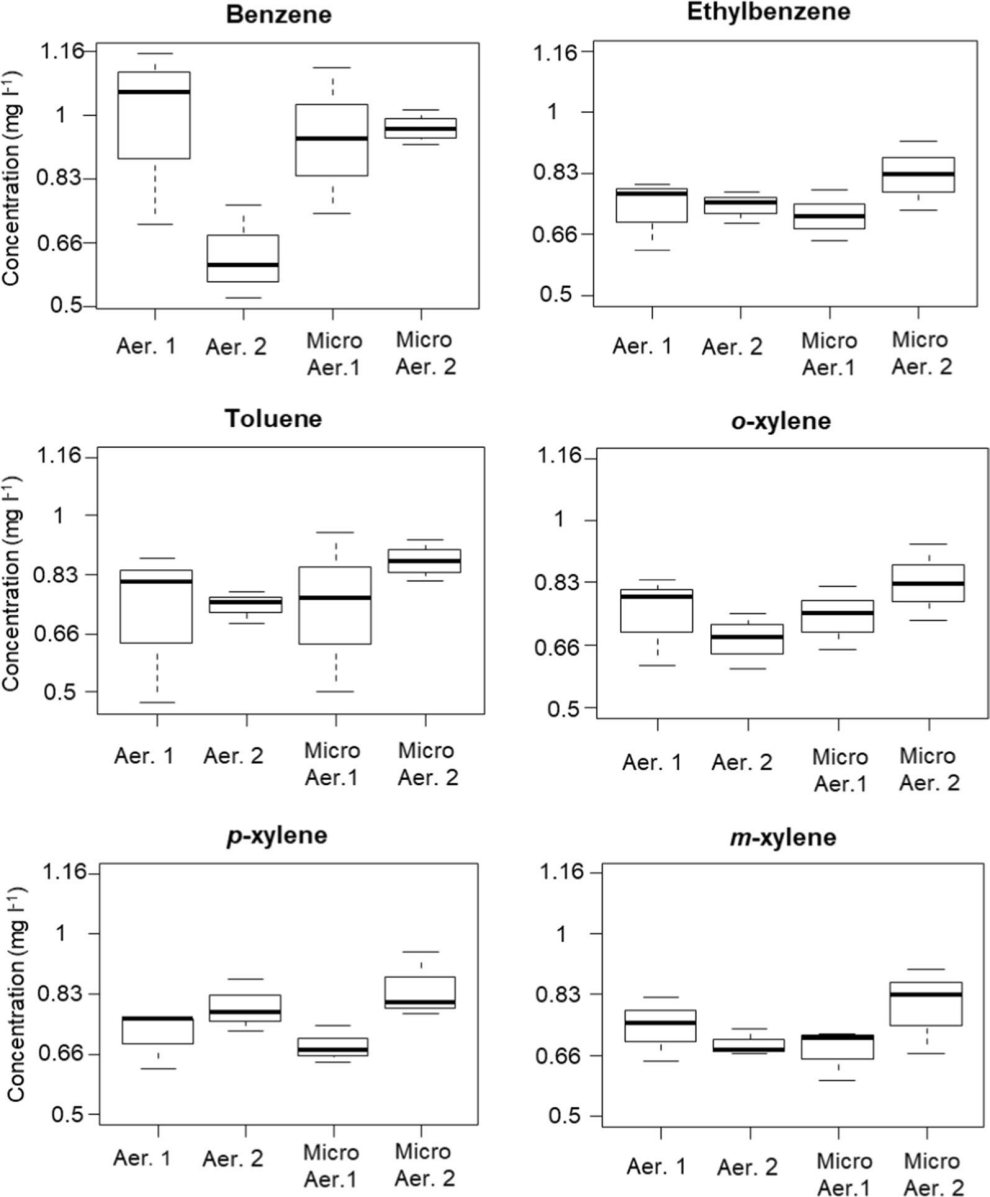
Boxplots showing median BTEX concentrations measured at the 168th hour of the first and second aerobic and micro-aerobic BTEX degradation experiments. Aer 1 and Micro Aer 1 median BTEX concentration values from the first aerobic and micro-aerobic experiments (microcosm experiment No 2); Aer 2 and Micro Aer 2 median BTEX concentration values from the second experiment (microcosm experiment No 3)

In the case of the tested organism, no statistically significant difference was observed between the BTEX mixture degradation capability under aerobic and micro-aerobic conditions (microcosm experiment No 2, Fig. 3; Additional file 1: Table S2). Moreover, at the 168th hour of incubation the degree of aerobic and micro-aerobic BTEX degradation between the two independent repetitions (microcosm experiment No 2 vs. 3) did not differ significantly either (p = 0.092), meaning that the amount and age of the inoculum presumably did not influence significantly the dynamics of BTEX biodegradation by *V. paradoxus* strain BFB1_13 (Fig. 4; Additional file 1: Table S3).

Over 2 weeks, for all replicates and oxygenation conditions, BTEX concentration reductions by *V. paradoxus* strain BFB1_13 varied from 91%, 96%, 73%, 85%, 71% and 62% to complete degradation at least in one of the replicates (100% conc. reduction) in the case of benzene, toluene, ethylbenzene, *o*-xylene, *m*-xylene and *p*-xylene, respectively. Based on the abovementioned results it can be assumed that *V. paradoxus* strain BFB1_13 degraded BTEX in the following order benzene, toluene and *o*-xylene, and ethylbenzene, *m*-xylene and *p*-xylene.

In the negative controls, the abiotic loss of benzene and toluene was negligible, however ethylbenzene, *o*-, *m*- and *p*-xylene showed a 36.5%, 32.7%, 41.4% and 41.4% concentration reduction, respectively. The very same abiotic loss was recorded for microcosm experiment No 1, No 2 and No 3 (data not shown).

#### The BTEX mixture biodegradation efficiency of the SBP-encapsulated culture

**Fig. 5:**
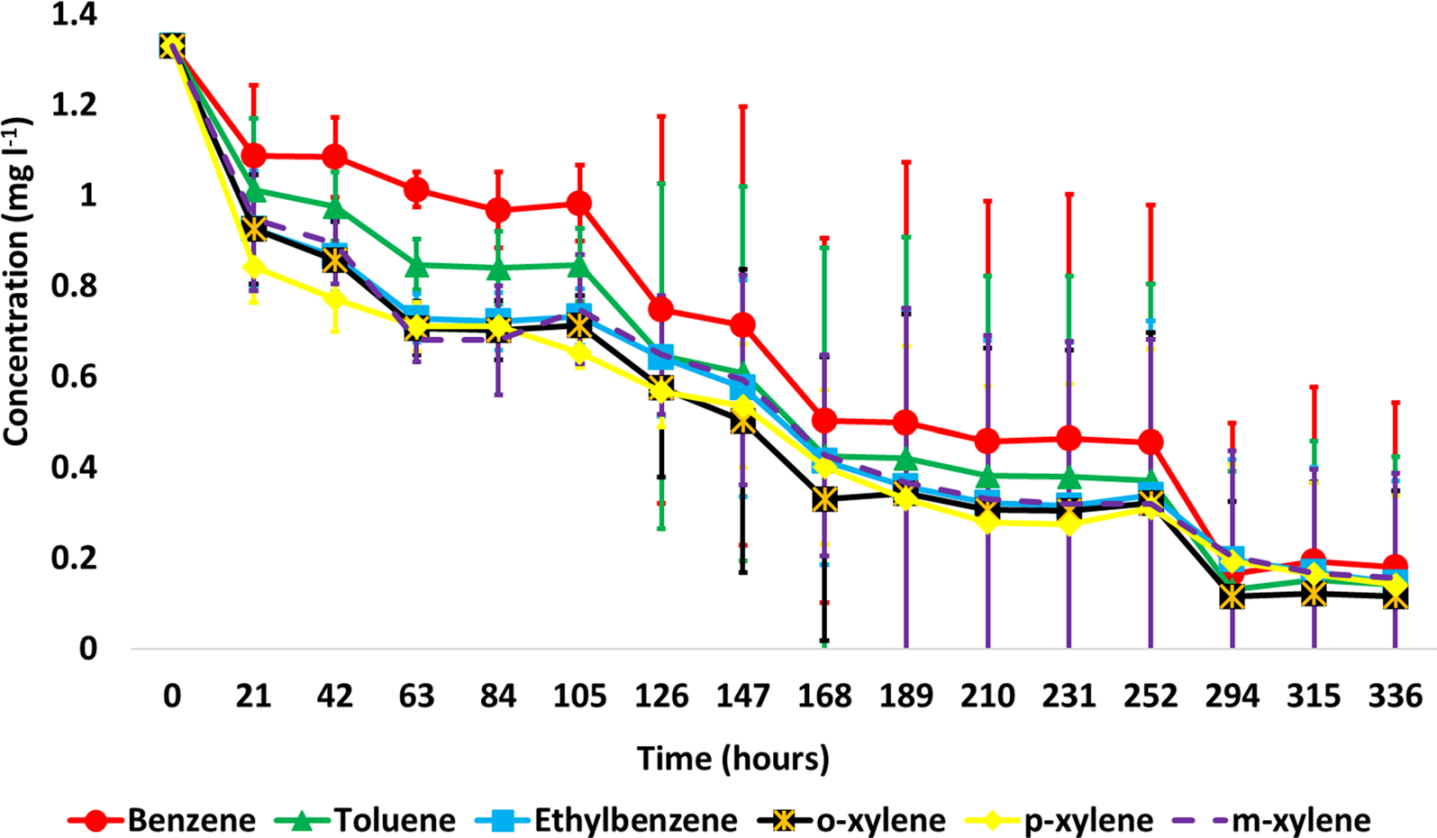
BTEX mixture biodegradation capacity of the SBP-encapsulated *V. paradoxus* strain BFB1_13

Similar to the results mentioned earlier, the SBP-encapsulated bacterial inoculum was also capable of degrading all six BTEX compounds. However, the BTEX degradation did not occur with the same efficiency in all the four replicates, high deviations were recorded (Fig. 5). At certain intervals, the capsules’ membrane slightly cracked resulting in the diffusion of bacterial cells into the medium. Once cells escaped from the capsules the rate of BTEX degradation soared. Bacterial leakages from the capsules could be observed visually, since the turbidity of the solution increased and bacterial flakes appeared in the bulk solution. The fastest BTEX degradation occurred in the case of the third replicate; after 126 h of incubation the initial concentration of benzene, toluene, ethylbenzene, *o*-, *m*- and *p*-xylene decreased by 89%, 91%, 56%, 72%, 61% and 46%, respectively. In the meantime, in the case of other replicates, where the cracking of the SBP capsules and the release of bacteria had not yet been observed, the concentration reduction of BTEX ranged between 20 and 32% for benzene, 24–43% for toluene, 30–52% for ethylbenzene, 30–56% for *o*-xylene, 30–56% for *m*-xylene and 30–60% for *p*-xylene. After 189 h of incubation, in the case of the first and third replicates the concentration of all BTEX decreased to almost zero. In the case of the fourth replicate notable BTEX biodegradation started to occur only after 294 h of incubation; by the end of the experiment the concentration of BTEX reduced to zero also in the case of this replicate. In the second replicate after 336 h of incubation only 26.6%, 36.6%, 43.9%, 45.5%, 39.5% and 49.1% of benzene, toluene, ethylbenzene, *o*-, *m*- and *p*-xylene had been eliminated, respectively; no cracking of the capsule and release of bacteria was observed.

By the end of the experiment, in the control samples the abiotic loss of BTEX compounds was negligible, suggesting that the capsule did not adsorb the BTEX (data not shown).

### Results of the whole genome sequencing and analysis

General features of the WGS sequencing of strain BFB1_13 are summarized in Table 1. The size of the recovered genome was 9,581,132 bp with a G+C content of 68.86%. The total number of coding sequences was 9586. Previous phylogenetic analysis based on *16S rRNA* gene sequences indicated that strain BFB1_13 belongs to the genus *Variovorax* and is most closely related to *V. paradoxus* NBRC 15149 and *V. boronicumulans* BAM-48, showing 99.2% *16S rRNA* gene sequence similarity to each (Additional file 1: Fig. S3). Based on whole genome sequences, the average nucleotide identity and in silico DNA-DNA hybridization values were 85.1% and 85.7 (ANI) and 30.3% and 34.2% (dDDH) for strains NBRC 15149 and BAM-48, respectively. The number of protein coding sequences of strain BFB1_13 was remarkably higher than that of the closest relatives, 6213 CDS for strain NBRC 15149 and 6198 CDS for strain BAM-48, indicating that presumably strain BFB1_13 has a more diverse metabolic capacity. On the other hand, total *rRNA* and *tRNA* gene numbers of strain BFB1_13 are relatively similar with those of strains NBRC 15149 (3 and 59) and BAM-48 (3 and 50).

**Table 1:** General features of the genome *Variovorax paradoxus* strain BFB1_13

Characteristic	Value
checkM* Completeness (%)	97.86 (9 markers are missing)
checkM contamination (%)	1.08 (5 markers are duplicated)
Size (bp)	9,581,132
G + C content (%)	68.86
Total number of genes	9668
Number of protein coding sequences	9586
Number of pseudogenes	11
Average gene length (bp)	928
rRNA (16S, 23S, 5S)	4
tRNA genes	51
Predicted transposase genes	22
Monooxygenase genes	32
Dioxygenase genes	59
GenBank accession number (NCBI)	JAEVYQ000000000

*CheckM embedded in MicroScope platform is an automated method for assessing the quality of a microbial genome regarding completion and contamination (Parks et al. 2015)

Strain BFB1_13 harbors genes encoding 32 monooxygenases and 60 dioxygenases that may be involved in the biodegradation of BTEX, terephthalate, benzoate/toluate, phenol, naphthalene, biphenyl and other hydrocarbons (Additional file 1: Table S4). Five catechol 2,3-dioxygenase genes (*xylE*) could be identified in the genome, which showed the highest amino acid sequence homology to *Pseudomonas putida* encoded *xylE* genes (Additional file 1: Table S5).

#### Genome-based prediction of putative BTEX degradation pathways of strain BFB1_13

Previously it has been shown that BTEX compounds can be metabolized through various metabolic pathways depending on the microorganisms (detailed below). Catabolic genes and pathways involved in biodegradation of BTEX were bioinformatically predicted (Additional file 1: Table S5).

##### Benzene biodegradation

Typically, benzene biodegradation can occur through two primary pathways catalyzed by either benzene 1,2-dioxygenase or phenol/toluene-(3/4)-monooxygenase, leading to the formation of cis-dihydrobenzenediol or phenol in the first oxidation step, respectively (Zamanian and Manson 1987; Tao et al. 2004).

**Fig. 6:**
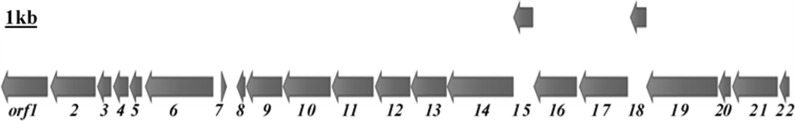
Physical map of the complete toluene 4-monooxygenase (T4MO) and the first phenol-2-hydroxylase (I. P2H) encoding gene clusters followed by genes involved in the “lower”-pathway of toluene/*p*-xylene metabolism. The putative functions of enzymes encoded by the genes were predicted as follows: *orf1* T4MO subunit *tmoF*, *orf2* T4MO subunit *tmoE*, *orf3* T4MO subunit *tmoD*, *orf4* T4MO subunit *tmoC*, *orf5* T4MO subunit *tmoB*, *orf6* T4MO subunit *tmoA*; *orf7* hypothetical protein, *orf8* 2-hydroxy-muconate tautomerase, *orf9* 4-oxalocrotonate decarboxylase, *orf10* 2-hydroxy-2-oxovalerate aldolase, *orf11* acetaldehyde-CoA dehydrogenase, *orf12* oxidoreductase, short chain dehydrogenase, *orf13* 2-keto-4-pentenoate hydratase*, orf14* 2-hydroxymuconic semialdehyde dehydrogenase, *orf15* uncharacterized protein, *orf16* catechol 2,3-dioxygenase (*xylE*), *orf17* P2H P5 protein (*dmpP*), *orf18* P2H P4 protein (*dmpO*), *orf19* P2H P3 protein (*dmpN*), *orf20* P2H P2 protein (*dmpM*), *orf21* P2H P1 protein (*dmpL*), *orf22* P2H P0 protein (*dmpK*)

No genes annotated as encoding for benzene monooxygenase or benzene 1, 2-dioxygenase were found in the genome of strain BFB1_13. However, a complete catabolic gene cluster encoding toluene 4-monooxygenase (T4MO) was detected. Five hundred bp downstream of the T4MO a complete phenol 2-hydroxylase encoding gene cluster (P2H, I. P2H cluster), followed by all the biodegradative genes involved in the “lower”-pathway of toluene/*p*-xylene biodegradation to pyruvate via 4-methylchatechol, could be detected as well (Fig. 6). This observation suggested that in strain BFB1_13, benzene is likely to be metabolized through the phenol degradation pathway, using T4MO for the activation of the aromatic ring (Additional file 1: Table S5).

It has to be noted that although the genome of strain BFB1_13 harbors only one single T4MO encoding gene, two additional phenol-hydroxylase encoding gene clusters were identified (Additional file 1: Tables S5 and S6). All P2H gene clusters are followed by *xylE,* catechol 2, 3-dioxygenase encoding genes, and further genes which products are involved in the “lower” degradation pathway of toluene and xylenes. While, the II. P2H is situated 38 kb downstream, the III. P2H is situated 0.6 Mb upstream of the I. P2H encoding gene cluster. Although, the II. P2H gene cluster lacked *dmpK* (P0) and *dmpL* (P1) subunits of the complete phenol hydroxylase-encoding gene operon, amino acid sequence homology between the existing subunits of the I. and II. P2H was 100%. In contrast, subunits of the III. phenol hydroxylase operon (III. P2H) showed only 42.3 to 71.6% amino acid sequence homology to the subunits of the previous two. Moreover, only the III. P2H encoding gene cluster was followed, amongst other “lower”-pathway biodegradative enzymes encoding genes, by 2-hydroxymuconate-6-semialdehyde hydrolase encoding gene, which is principally involved in the biodegradation of *o*- and *m*-xylene.

##### Toluene biodegradation

Five aerobic toluene biodegradation pathways have been reported so far. Toluene biodegradation can be initiated by the oxidation of the methyl group (Shaw and Harayama 1992), ring mono-oxidation at position 2, 3 or 4 (Olsen et al. 1994; Shields et al. 1995; Yen et al. 1991), or ring 2, 3-dioxygenation (Wackett et al. 1988).

Bioinformatic analysis of the toluene catabolic genes revealed that strain BFB1_13 is likely capable of transforming toluene using two different pathways. In the first case, toluene is activated by T4MO and the resulting *p*-cresol is further transformed by P2H to 4-methylcatechol. Thereafter catechol is cleaved by catechol 2, 3-dioxygenase (*xylE*) and further converted to pyruvate by the aid of “lower”-pathway degrading enzymes shown in Fig. 6 (*orfs 8–14*). According to the second predicted pathway, toluene biodegradation is initiated by the xylene-monooxygenase (*xylM*), oxidation of the methyl group. The resulting benzyl alcohol is converted to benzaldehyde and subsequently to benzoate via benzyl alcohol dehydrogenase and benzaldehyde dehydrogenase, respectively. Next, benzoate enters to the benzoate degradation pathway and leads to the formation of pyruvate. A x*ylM* gene*,* whose product in general oxidizes toluene and xylenes to (methyl)benzyl alcohols, was found 1.15 kb upstream of the T4MO encoding gene cluster of strain BFB1_13. All further genes which products are involved in the transformation of benzyl alcohol to pyruvate could also be identified in the genome, however, present in different gene clusters. This finding indicated that the expression of these genes may be separately and independently regulated (Additional file 1: Table S5).

The presence of T4MO encoding gene in the genome led to the assumption that toluene metabolism could occur through another pathway, such as formation of *p*-cresol and its subsequent conversion to 4-hydroxy-benzaldehyde and 4-hydroxy-benzoate catalyzed by 4-cresol dehydrogenase (*pch*F) and 4-hydroxybenzaldehyde dehydrogenase (*pch*A), respectively. Since 4-cresol dehydrogenase encoding gene was not detected in the genome, this degradation pathway was either incomplete in the recovered genome or generally incomplete in strain BFB1_13.

No genes encoding for toluene 2 (or 3)-monooxygenase or toluene 2, 3-dioxygenase were detected in the genome of strain BFB1_13.

##### Biodegradation of xylenes

Microcosm experiments indicated that strain BFB1_13 is capable of degrading all the three xylene isomers (*o*-, *m*- and *p*-xylene, Figs. 2B, 3 and 4).

In general, the biodegradation of xylenes is initiated through the oxidation of a methyl substituent to methylbenzyl-alcohol by xylene monooxygenase (*xylM*) or the direct oxidation of the aromatic ring by xylene dioxygenase (Cao et al. 2009; Jang et al. 2005; Yu et al. 2001). In the xylene monooxygenase pathway, the methylbenzyl-alcohol is subsequently converted to methyl benzaldehyde, methyl benzoate and 1, 2-dihydroxy-methyl-cyclohexa-3,5-diene-carboxylate by benzylalcohol dehydrogenase (*xylB*), benzaldehyde dehydrogenase (*xylC*) and benzoate 1, 2-dioxygenase (*xylX*), respectively. Eventually, the latter is transformed to 3 or 4-methylcatechol by dihydroxycyclohexadiene carboxylate dehydrogenase (*xylL*). The resulting methylcatechol is cleaved by catechol 2, 3-dioxygenase (*xylE*) and is subsequently converted to pyruvate through a series of enzymatic reactions.

All genes whose products are involved in the biodegradation of xylenes, through the oxidation of a methyl substituent (*xylMBCXL*), were detected in the genome of strain BFB1_13 (Fig. 6, *orfs 8-14,* Additional file 1: Table S5). However, these genes were present in different gene clusters, indicating that they may separately and independently be regulated. Therefore, xylene biodegradation by strain BFB1_13 most probably occurred through the monooxygenation of a methyl substituent. The biotransformation of *p-*xylene most probably led to the formation of 4-methylcatechol, which after ring cleavage was further converted to pyruvate using “lower”-pathway degrading enzymes (Fig. 6, *orfs 8-14*). On the other hand, presumably *o*- and *m*-xylene were converted to 3-methylcatechol, which after ring fission was further converted to pyruvate through the activity of 2-hydroxymuconate-6-semialdehyde hydrolase, 2-keto-4-pentenoate hydratase and 4-hydroxy 2-oxovalerate aldolase. Gene encoding 2-hydoxymuconate-6-semialdehyde hydrolase is present in the genome in two copies (Additional file 1: Table S5).

The genome annotation of strain BFB1_13 failed to uncover xylene dioxygenase encoding genes.

##### Ethylbenzene biodegradation

Two aerobic ethylbenzene biodegradation pathways have been reported: (i) direct aromatic ring oxidation by ethylbenzene dioxygenase (Gibson et al. 1973), and (ii) the ethyl group oxidation by naphthalene dioxygenase (Lee and Gibson 1996). The naphthalene dioxygenase catalyzed ethylbenzene biodegradation leads to the formation of styrene or 1-phenethyl alcohol. The latter, through the formation of acetophenone, can be converted to either 2-hydroxy-acetophenone or benzoylacetate.

Metabolic analysis indicated that the genome of strain BFB1_13 does not contain ethylbenzene dioxygenase related or genes homologues with ethylbenzene dioxygenase, nor acetophenone carboxylase involved in acetophenone transformation to benzoylacetate. However, genes encoding for naphthalene dioxygenase, as well as the majority of enzymes involved in the degradation of styrene to fumarate (6 key functional genes out of 8) were detected (Additional file 1: Table S5). Therefore, it can be assumed that ethylbenzene biodegradation by strain BFB1_13 could occur through the oxidation of the ethyl-group by naphthalene dioxygenase leading to the formation of both styrene and 2-hydroxy-acetophenone.

## Discussion

In this comprehensive study a *Variovorax paradoxus* strain was investigated to assess its applicability in the biological removal of BTEX contamination. The subject of our study, *V. paradoxus* strain BFB1_13 was capable of degrading all six BTEX compounds under both, aerobic and micro-aerobic/oxygen-limited conditions (Figs. 2, 3, 4). It has to be highlighted that in the literature only a handful of isolates have been reported capable of degrading alone all six BTEX: *Pseudoxanthomonas spadix* BDa-59 (Choi et al. 2013), *Paraburkholderia aromaticivorans* BN5 (Lee et al. 2019), *Dechloromonas* sp. RCB (anaerobic biodegradation pathway, Chakraborty et al. 2005), *Ralstonia* sp. PHS1 (Sung-Kuk and Lee 2002), *Ralstonia pickettii* PKO1 (Leahy et al. 2003), *Rhodococcus* sp. ZJUT312 (You et al. 2018); *Rhodococcus rhodochrous* (Deeb and Alvarez-Cohen 1999); and *Pseudomonas putida* YNS1 (You et al. 2012).

In the BTEX containing microcosms, the order of biodegradation by strain BFB1_13 was benzene (B), toluene (T) and *o*-xylene (*o*X), ethylbenzene (EB), *m*- and *p*-xylene (*m*X and *p*X). The sequence of BTEX biodegradation followed the order of water solubility of the tested compounds measured at 25 °C (benzene 1.77 g l^−1^, toluene 0.52 g l^−1^, *o*-xylene 0.18 g l^−1^, ethylbenzene 0.17 g l^−1^, *m*- and *p*-xylene 0.16 g l^−1^, Kim et al. 2019). The same preference towards BTEX, and the same order of degradation was reported in the case of *Ralstonia* sp. PHS1 too. However, different biodegradation orders were found in the case of *P. aromaticivorans* BN5 (EB, T < *o*X < *m*X, *p*X < B), *Rhodococcus* sp. ZJUT312 (*m*X, *p*X < B < T < EB < *o*X) and *P. putida* YNS1 (*o*X < *p*X < *m*X < B < EB < T). It has to be added that different initial BTEX concentrations were applied in the last three cases: *P. aromaticivorans* BN5—30 mg l^−1^; *Rhodococcus* sp. ZJUT312—1 mmol l^−1^; *P. putida* YNS1—100 mg l^−1^.

The results of the microcosm experiment No 1 suggested that the presence of ethylbenzene, *m*- and *p*-xylene may inhibit the BTEX biodegradation capability of *V. paradoxus* strain BFB1_13 (Fig. 2A and)B. The assumed inhibitory nature of these compounds on BTEX biodegradation has already been demonstrated by Deeb and Alvarez-Cohen (1999) and Sung-Kuk and Lee (2002).

No statistically significant difference was observed between aerobic and micro-aerobic BTEX degradation of strain BFB1_13. This finding promotes the applicability of the strain in the biodegradation of BTEX under oxygen concentrations as low as 0.5 mg l^−1^. Most probably, the ex or in situ use of strain BFB1_13 for BTEX removal would remarkably reduce the costs of interventions, since no substantial oxygen supply or additional operational costs related to aeration are required to achieve high degradation efficiencies and to maintain the most efficient aerobic degradation routes (not to mention that uncontrolled aeration could lead to the transfer of volatile organic compounds, such as carcinogenic BTEX, to the atmosphere). This can be particularly advantageous during the application of strain BFB1_13 for in situ bioremediation (bioaugmentation) of petroleum hydrocarbon contaminated shallow groundwaters, where the concentration of oxygen is generally low, found in the hypoxic range (≤ 2 mg l^−1^; Benedek et al. 2016; Marić et al. 2020). The fact that strain BFB1_13 showed a notable growth also at temperature levels as low as 10 or 15 °C—checked in nutrient-broth—further justifies its use for shallow groundwater remediation. It has to be mentioned that in general in central Europe the mean temperature of shallow groundwaters is about 10 °C, as well as ranges between 10 and 14 °C, respectively (Ministry for Environment and Water 2006; Tissen et al. 2019).

Strain BFB1_13 showed notable biofilm-forming capabilities too (Fig. 1). The prolific biofilm-forming ability of microbial cells may facilitate the biodegradation of petroleum hydrocarbons. EPS containing surfactants may also aid solubilization of hydrophobic compounds which would otherwise be inaccessible to microorganisms (Mitra and Mukhopadhyay 2016; Iwabuchi et al. 2002). According to Omarova et al. (2019), microbial biofilms aid in the stabilization of dispersed oil droplets, indirectly accelerating the process of oil biodegradation. In the study of Dasgupta et al. (2013) and Shimada et al. (2012) biofilm-based batch cultures were more efficient in hydrocarbon degradation than planktonic cultures. Based on the whole genome sequence analysis, *V. paradoxus* strain BFB1_13 harbors the following genes involved in biofilm development and extracellular matrix production: *clfA*—encoding clumping factor A (locus tag VPARBFB13_v1_2150015; Rahimi et al. 2016); *pilA (*VPARBFB13_v1_1840001) and *pilB* (VPARBFB13_v1_2300035) encoding type IV pili synthesis involved in biofilm formation (Konto-Ghiorghi et al. 2009); *flgK*—encoding flagella expression (VPARBFB13_v1_170027; O’Toole and Kolter 1998).

Microcosm experiments containing SBP-encapsulated bacterial cultures showed that bacteria released from the cracked capsules were metabolically active and degraded BTEX even after being in an encapsulated state of 10 days (Fig. 5). This finding may indicate that the capsule, as well as the 2% R2A medium ensured the survival and growth of the bacterium, which through the microfiltration membrane could gradually adapt to the presence of BTEX. Once bacterial cells had been released, the contact area between the BTEX and the biomass (the bioavailability of BTEX) increased leading to the swift and almost complete degradation of the compounds within 40 h. It is noteworthy that the SBP encapsulation technology has already been applied successfully for the treatment of industrial (olive mill wastewater) and municipal wastewater and for the biodegradation of hydrophobic and hydrophilic compounds, such as ethynylestradiol (EE2) and phenolic compounds (Azaizeh et al. 2015; Menashe and Kurzbaum 2014; Kurzbaum et al. 2020a, b; Miller et al. 2020). It has also been demonstrated that the hydrophilic cellulose acetate microfiltration membrane of the SBP capsule allows the traversing of both hydrophilic (phenols) and hydrophobic molecules (EE2). BTEX are relatively hydrophobic in nature, thus their diffusion through the membrane, to some extent, could be expected. Moreover, it has also been found that in phenol rich test solutions (1000 mg l^−1^), SBP encapsulated bacterial cells performed better compared to suspended cultures (Kurzbaum et al. 2020a). In this study, no remarkable difference was observed between the BTEX biodegradation rate of encapsulated and planktonic cultures. In both settings complete BTEX-biodegradation occurred after at least 168 h of incubation.

By using *V. paradoxus* BFB1_13 containing SBP capsules the establishment of in situ semipermeable reactive biobarriers (Careghini et al. 2013) would be possible for the containment and decontamination of BTEX-polluted shallow groundwaters, generally having low temperature and oxygen concentration. Miller et al. (2020) already have proven the applicability of the SBP-technology for the removal of 3-chlorophenol (concentration range between 350 and 500 mg l^−1^) in a pilot permeable reactive biobarrier system by using encapsulated *Pseudomonas putida* (Miller et al. 2020). The biggest advantage of the SBP technology is that the key element, the microfiltration membrane, with unique mesh-like porous structural properties, protects the introduced bacterial culture by creating a semi-confined aqueous environment. The semipermeable membrane does not allow predators or rival microorganisms to get inside of the capsule. However, on the other hand, it allows the diffusion of pollutants or other nutrients, as well as oxygen into the internal environment. Similarly, through the pores of the capsule, the release of CO_2_ originating from the microbial degradation of a given pollutant is also possible (Menashe et al. 2020b). Additionally, the SBP technology allows the simultaneous encapsulation of easily metabolizable nutrients together with the biomass, which could facilitate the growth and increase the survival and adaptation of the encapsulated and activated biomass introduced into the contaminated environment (Kurzbaum et al. 2020b; Miller et al. 2020).

It was predicted that strain BFB1_13 started the biodegradation of benzene with the formation of phenol by toluene-4-monooxygenase, which is then transformed to catechol by phenol-2-hydroxylase. Ring fission of the catechol intermediate was catalyzed by catechol 2, 3-dioxygenase. The same degradation pathway was predicted also for toluene. The very same benzene metabolic pathway has been described for *Paraburkholderia aromaticivorans* BN5 and *Pseudoxanthomonas spadix* BDa-59 by Lee et al. (2019) and Choi et al. (2013), respectively. However, in the case of strains BN5 and BDa-59, toluene metabolism occurred through the formation of *p*-hydroxybenzyl alcohol, *p*-hydroxybenzaldehyde and *p*-hydroxybenzoate. This toluene degradation pathway was not possible by strain BFB1_13 since it lacked *PchCF* and *PchA* genes. Consequently, this brings up the question “how toluene biodegradation could be initiated by T4MO, if further genes leading to the formation of *p*-hydroxybenzoate were not in the genome?” One possible explanation could be that toluene was converted to *p*-cresol by T4MO which was further converted to 4-methylcatechol by phenol-2-hydroxylase (Ma et al. 2013). The same toluene biodegradation pathway was described for *Pseudomonas stutzeri* OX1 (Parales et al. 2008). It has to be noted that presumably toluene degradation could also occur through the activity of *xylM,* leading to the formation of benzyl alcohol, benzaldehyde and benzoate. The predicted xylene and ethylbenzene metabolic pathways in the case of strain BFB1_13 have already been described for strains BN5 and BDa-59 mentioned before. In addition, the proposed ethylbenzene oxidation by strain BFB1_13 through styrene formation was proved in *Pseudomonas* sp. strain NCIB 9816-4 (Lee and Gibson 1996).

In conclusion, this study describes for the first time a *V. paradoxus* isolate capable of degrading all six BTEX compounds under both, aerobic and micro-aerobic/oxygen-limited conditions. BTEX biodegradation genes have been annotated and degradation pathways have been predicted, however to fully support the suggested biodegradation pathways further experiments are needed. *V. paradoxus* strain BFB1_13 can most probably be applied in the remediation of BTEX contaminated sites, especially BTEX-polluted shallow groundwaters with low oxygen concentration and temperature. The optimal cultivation conditions necessary for the production of the inoculum were determined. The application of SBP encapsulated *V. paradoxus* BFB1_13 for the development of reactive biobarriers for the containment and decontamination of BTEX contaminated aquifer is highly recommended. The application of SBP encapsulated *V. paradoxus* strain BFB1_13 for BTEX decontamination would have the following main advantages: (i) no washout or dilution of the inoculum—higher retention time in the contaminant plume; (ii) physical protection that increases the tolerance and adaptation of the microbial culture; (iii) microscopic cracks in the SBP capsules’ membrane allow the diffusion of cells out from the capsule and continuous inoculation of the nearby environment; the release of already adapted *V. paradoxus* BFB1_13 cells may amplify the process of biodegradation and could increase the physical dimensions of the biobarrier.

*Variovorax paradoxus* strain BFB1_13 alone or in co-culture with other BTEX degrading bacterial isolates can be a new and efficient bioremediation agent for BTEX contaminated sites. Since *V. paradoxus* strain BFB1_13 showed low ANI (85.1% and 85.7%) and dDDH (30.3% and 34.2%) values with the closest relatives—determined on the basis of *16S rRNA* gene sequence comparisons—lower than the 95–96% ANI and 70% dDDH thresholds for species delineation (Kim et al. 2014; Rosselló-Mora and Amann 2015), there is a high chance that strain BFB1_13 is actually a new species of the genus *Variovorax*. However, to fully support this assumption a polyphasic taxonomic study, including phenotypic and chemotaxonomic characterizations would be needed (Tindall et al. 2010), which goes beyond the scope of this paper.

## Supplementary Information

**Additional file 1.** Additional Figures and Tables.

## Data Availability

Data generated or analyzed during this study are included in this published article (and its additional files). The genome sequence of *V. paradoxus* strain BFB1_13 has been deposited to GenBank (NCBI) under the accession number JAEVYQ000000000. *V. paradoxus* strain BFB1_13 is deposited in the National Collection of Agricultural and Industrial Microorganisms (NCAIM, Budapest, Hungary) under the accession number NCAIM B.02666.
