# Supplementary material for: Potential of Variovorax paradoxus isolate BFB1_13 for bioremediation of BTEX contaminated sites
Source: AMB Express. 2021 Sep 6;11:126. doi: 10.1186/s13568-021-01289-3 (PMC8421498; doi:10.1186/s13568-021-01289-3)
Supplement: Supplementary file 1 — Additional file 1. Additional Figures and Tables. [file 13568_2021_1289_MOESM1_ESM.pdf]

**Journal Name: AMB Express**

# Manuscript Title: Potential of *Variovorax paradoxus* isolate BFB1_13 for bioremediation of BTEX contaminated sites

Tibor BENEDEK[a*], Flóra SZENTGYÖRGYI[a], Veronika GERGÓCS[b], Ofir MENASHE[c,d], Perla Abigail FIGUEROA GONZALEZ[e], Alexander J. PROBST[e], Balázs KRISZT[f], András TÁNCSICS[a]

[a]*Hungarian University of Agriculture and Life Sciences, Institute of Aquaculture and Environmental Safety, Department of Molecular Ecology, Gödöllő, H-2100, Páter K. u. 1, Hungary;*

[b]*Institute for Soil Sciences, Budapest, H-1022, Herman Ottó út 15, Hungary*

[c]*Water Industry Engineering Department, Achi Racov School of Engineering, Kinneret Academic College on the Sea of Galilee, D.N. Emek Ha'Yarden 15132, Israel*

[d]*BioCastle Water Technologies Ltd., Tzemah, Israel*

[e]*Group for Aquatic Microbial Ecology, Environmental Microbiology and Biotechnology, Faculty of Chemistry, University of Duisburg-Essen, Essen, Universitätsstr. 5, 45141 Essen, Germany*

[f]*Hungarian University of Agriculture and Life Sciences, Institute of Aquaculture and Environmental Safety, Department of Environmental Safety, Gödöllő, H-2100, Páter K. u. 1, Hungary;*

*Corresponding author: Tel.: +36-(28)-522-000 ext. 1611

E-mail: benedek.tibor@uni-mate.hu

Number of figures: 3

Number of tables: 6

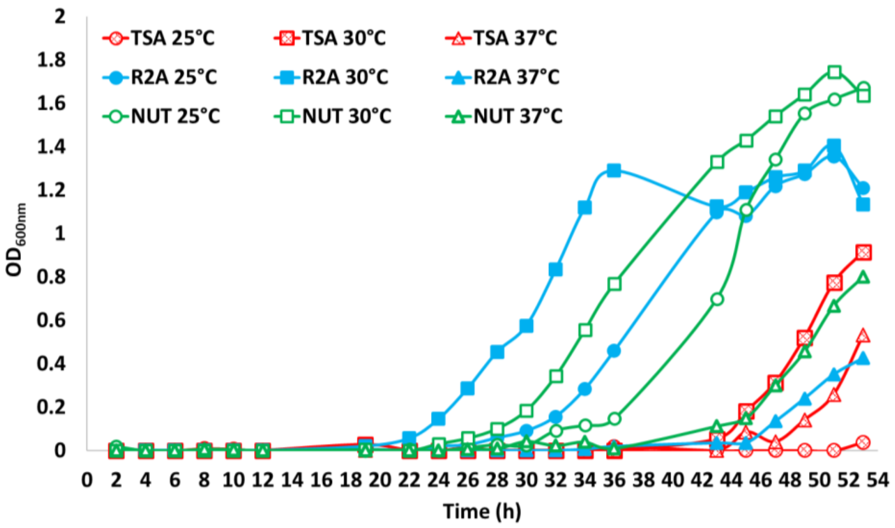


**Fig. S1 Optimal growth media and temperature for the cultivation of *V. paradoxus* strain BFB1_13 assessed at pH 7**

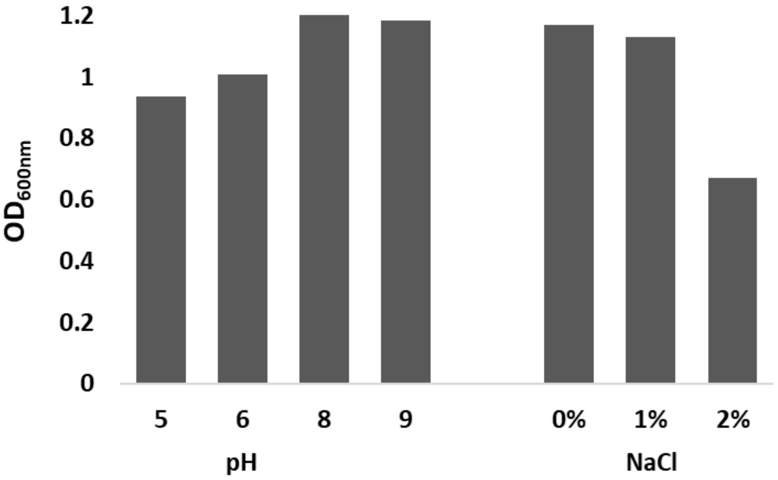


**Fig. S2 pH and NaCl tolerance of V. paradoxus strain BFB1_13 assessed after 48 hours of incubation in nutrient-broth at 30 ºC.**

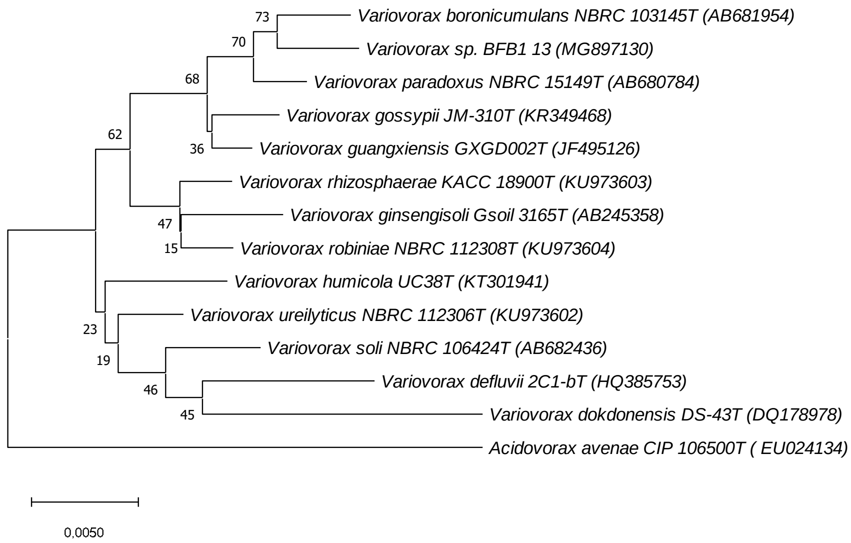


**Fig. S3 Evolutionary relationships of *Variovorax* type species**

The evolutionary history was inferred using the Neighbor-Joining method [1]. The optimal tree with the sum of branch length = 0.10070672 is shown. The percentage of replicate trees in which the associated taxa clustered together in the bootstrap test (10000 replicates) are shown next to the branches [2]. The tree is drawn to scale, with branch lengths in the same units as those of the evolutionary distances used to infer the phylogenetic tree. The evolutionary distances were computed using the Maximum Composite Likelihood method [3] and are in the units of the number of base substitutions per site. This analysis involved 14 nucleotide sequences. All ambiguous positions were removed for each sequence pair (pairwise deletion option). There were a total of 1401 positions in the final dataset. Evolutionary analyses were conducted in MEGA X [4].

1. Saitou N. and Nei M. (1987). The neighbor-joining method: A new method for reconstructing phylogenetic trees. *Molecular Biology and Evolution* 4:406-425.

2. Felsenstein J. (1985). Confidence limits on phylogenies: An approach using the bootstrap. *Evolution* 39:783-791.

3. Tamura K., Nei M., and Kumar S. (2004). Prospects for inferring very large phylogenies by using the neighbor-joining method. *Proceedings of the National Academy of Sciences* (*USA*) 101:11030-11035.

4. Kumar S., Stecher G., Li M., Knyaz C., and Tamura K. (2018). MEGA X: Molecular Evolutionary Genetics Analysis across computing platforms. *Molecular Biology and Evolution* 35:1547-1549.

**Table S1:** Composition of the used growth media

| Component (g) | Growth Media | | |
|---|---|---|---|
| | Nutrient | R2A | TSA |
| **NaCl** | 5 | - | 5 |
| **Yeast Extract** | 2 | 0.5 | - |
| **Meat Extract** | 1 | - | - |
| **Tryptone** | - | - | 15 |
| **Casein Peptone** | 5 | 0.5 | - |
| **Soy Peptone** | - | - | 5 |
| **Soluble Starch** | - | 0.5 | - |
| **$K_2HPO_4$** | - | 0.3 | - |
| **$MgSO_4 \cdot 7\ H_2O$** | - | 0.05 | - |
| **Sodium-Pyruvate** | - | 0.3 | - |
| **D-Glucose** | - | 0.5 | - |
| **Casamino Acids** | | 0.5 | - |
| **$H_2O$** | 1000 | 1000 | 1000 |

**Table S2:**

Three types of models could be fitted to degradation processes of the six molecules. The table contains the name of the curve type and the coefficient estimates of the fitted models (±standard error). T-tests compared the coefficients between the two systems and p-values are displayed. The explanation of the parameters are the following:

Logistic function : $Y = p_1 + p_2/(1 + \exp(-p_3 * (X - p_4)))$

Saturation function: $Y = p_1 + p_2 * (1- \exp(-p_3 * X ))$

Linear function: Y = intercept + slope * X

| Compounds | Systems | Models | Parameters: | | | |
|---|---|---|---|---|---|---|
| | | | $p_1$ | $p_2$ | $p_3$ | $p_4$ |
| Benzene | Aerob | **logistic** | 0.66±0.37 | 7.25±0.85 | 0.56±0.16 | 9.69±0.53 |
| | Micro | **curve** | 0.45±0.41 | 7.11±0.82 | 0.55±0.15 | 9.01±0.53 |
| | t-test | p-value | 0.709 | 0.904 | 0.954 | 0.367 |
| Toluene | Aerob | **logistic** | 0.37±0.61 | 8.12±1.16 | 0.39±0.11 | 8.37±0.60 |
| | Micro | **curve** | 0.51±0.52 | 7.64±0.92 | 0.44±0.11 | 8.07±0.53 |
| | t-test | p-value | 0.86 | 0.746 | 0.738 | 0.711 |
| Ethyl- | Aerob | **saturation** | 0.61±0.29 | 7.56±1.55 | 0.07±0.03 | - |
| benzene | Micro | **curve** | 0.62±0.28 | 10.46±3.04 | 0.05±0.02 | - |
| | t-test | p-value | 0.972 | 0.398 | 0.587 | - |
| *p*-xylene | Aerob | **saturation** | 0.41±0.31 | 5.64±0.55 | 0.13±0.03 | - |
| | Micro | **curve** | 0.76±0.29 | 8.40±2.00 | 0.07±0.03 | - |
| | t-test | p-value | 0.408 | 0.188 | 0.161 | - |
| *m*-xylene | Aerob | **saturation** | 0.38±0.31 | 8.59±2.23 | 0.06±0.03 | - |
| | Micro | **curve** | 0.75±0.31 | 8.80±1.80 | 0.07±0.03 | - |
| | t-test | p-value | 0.397 | 0.94 | 0.861 | - |
| | | | slope | | | |
| *o*-xylene | Aerob | **linear** | 0.77±0.48 | | | |
| | Micro | **function** | 0.76±0.48 | | | |
| | t-test | p-value | 0.986 | | | |

**Table S3:**

Results from the Kruskal-Wallis tests comparing the concentration reduction values at the 168$^{th}$ hour for the first and second investigation (microcosm experiment Nº 2 vs. 3).

| | Chi-squared value | p-value |
|---|---|---|
| Benzene | 4.64 | 0.200 |
| Toluene | 3.00 | 0.392 |
| Ethylbenzene | 2.59 | 0.459 |
| *o*-xylene | 3.00 | 0.392 |
| *p*-xylene | 6.44 | 0.092 |
| *m*-xylene | 2.59 | 0.459 |

**Table S4:** Monooxygenase and dioxygenase genes encoded by *Variovorax paradoxus* strain BFB1_13

**Monooxygenases encoded by *V. paradoxus* strain BFB1_13**

| Locus tag (MaGe) | Gene | Length (aa) | Putative function |
|---|---|---|---|
| VPARBFB13_v1_5500013 | *oxyS* | 510 | Anhydrotetracycline 6-monooxygenase |
| VPARBFB13_v1_2430008 | _ | 545 | 4-hydroxyphenylacetate 3-monooxygenase |
| VPARBFB13_v1_3890036 | *nphA* | 528 | 4-nitrophenol 2-monooxygenase, oxygenase component |
| VPARBFB13_v1_2180002 | *phzS* | 417 | 5-methylphenazine-1-carboxylate 1-monooxygenase |
| VPARBFB13_v1_3360002 | *phzS* | 432 | 5-methylphenazine-1-carboxylate 1-monooxygenase |
| VPARBFB13_v1_6040005 | *nicC* | 381 | 6-hydroxynicotinate 3-monooxygenase |
| VPARBFB13_v1_2080059 | _ | 375 | Alkanesulfonate monooxygenase |
| VPARBFB13_v1_5810015 | _ | 358 | Alkanesulfonate monooxygenase |
| VPARBFB13_v1_3810102 | *ssuD* | 390 | Alkanesulfonate monooxygenase, FMNH(2)-dependent |
| VPARBFB13_v1_5170002 | *ssuD* | 388 | Alkanesulfonate monooxygenase, FMNH(2)-dependent |
| VPARBFB13_v1_790010 | *petC* | 263 | Ammonia monooxygenase gamma subunit |
| VPARBFB13_v1_4400034 | _ | 792 | Anthraniloyl-CoA monooxygenase |
| VPARBFB13_v1_330012 | _ | 95 | Antibiotic biosynthesis monooxygenase |
| VPARBFB13_v1_1620036 | _ | 116 | Antibiotic biosynthesis monooxygenase |
| VPARBFB13_v1_2040006 | _ | 107 | Antibiotic biosynthesis monooxygenase |
| VPARBFB13_v1_2960008 | _ | 100 | Antibiotic biosynthesis monooxygenase |
| VPARBFB13_v1_5520003 | _ | 112 | Antibiotic biosynthesis monooxygenase |
| VPARBFB13_v1_2640009 | _ | 395 | Sulfonate monooxygenase |
| VPARBFB13_v1_1040001 | *dmoA* | 486 | Dimethyl-sulfide monooxygenase |
| VPARBFB13_v1_2070004 | *dmoA* | 461 | Dimethyl-sulfide monooxygenase |
| VPARBFB13_v1_3740029 | *dmoA* | 502 | Dimethyl-sulfide monooxygenase |
| VPARBFB13_v1_3740039 | *dmoA* | 469 | Dimethyl-sulfide monooxygenase |
| VPARBFB13_v1_3740043 | *dmoA* | 458 | Dimethyl-sulfide monooxygenase |
| VPARBFB13_v1_5010008 | *dmoA* | 484 | Dimethyl-sulfide monooxygenase |
| VPARBFB13_v1_5010010 | *sfnG* | 376 | FMNH(2)-dependent dimethylsulfone monooxygenase |
| VPARBFB13_v1_370009 | *limB* | 390 | Limonene 1,2-monooxygenase |
| VPARBFB13_v1_1280009 | *pvdA* | 437 | L-ornithine N(5)-monooxygenase |
| VPARBFB13_v1_3050015 | _ | 389 | Luciferase-like monooxygenase |
| VPARBFB13_v1_3970001 | _ | 519 | Monooxygenase |
| VPARBFB13_v1_1840048 | *rutA* | 371 | Monooxygenase of the alternative pyrimidine degradation |
| VPARBFB13_v1_370007 | *scmK* | 451 | N-acetyl-S-(2-succino)cysteine monooxygenase |
| VPARBFB13_v1_5610004 | *scmK* | 444 | N-acetyl-S-(2-succino)cysteine monooxygenase |

| | | | |
|---|---|---|---|
| VPARBFB13_v1_5810014 | *scmK* | 456 | N-acetyl-S-(2-succino)cysteine monooxygenase |
| VPARBFB13_v1_3300031 | _ | 170 | Nitrilotriacetate monooxygenase component B |
| VPARBFB13_v1_350002 | _ | 321 | Nitronate monooxygenase |
| VPARBFB13_v1_5060008 | _ | 373 | Nitronate monooxygenase |
| VPARBFB13_v1_5640002 | _ | 320 | Nitronate monooxygenase |
| VPARBFB13_v1_60032 | _ | 93 | Phenol 2-monooxygenase P0 subunit |
| VPARBFB13_v1_1220004 | *sfnC* | 440 | putative FMNH2-dependent monooxygenase SfnC |
| VPARBFB13_v1_3740042 | *sfnC* | 394 | putative FMNH2-dependent monooxygenase SfnC |
| VPARBFB13_v1_110011 | *ygiN* | 103 | putative quinol monooxygenase YgiN |
| VPARBFB13_v1_720003 | _ | 485 | putative Unspecific monooxygenase |
| VPARBFB13_v1_2930007 | _ | 104 | Quinol monooxygenase YgiN |
| VPARBFB13_v1_420067 | *tmoD* | 103 | Toluene-4-monooxygenase, effector component |
| VPARBFB13_v1_420068 | *tmoC* | 112 | Toluene-4-monooxygenase, ferredoxin component |
| VPARBFB13_v1_420070 | *tmoA* | 497 | Toluene-4-monooxygenase, hydroxylase component subunit α |
| VPARBFB13_v1_420066 | *tmoE* | 329 | Toluene-4-monooxygenase, hydroxylase component subunit β |
| VPARBFB13_v1_420069 | *tmoB* | 91 | Toluene-4-monooxygenase, hydroxylase component subunit γ |
| VPARBFB13_v1_4490014 | *tsaM* | 357 | Toluene-4-sulfonate monooxygenase, iron-sulfur subunit TsaM1 |
| VPARBFB13_v1_3570073 | _ | 176 | Ubiquinone biosynthesis monooxygenase Coq7 |

**Dioxygenases encoded by *V. paradoxus* BFB1_13**

| Locus tag (MaGe) | Gene | Length (aa) | Putative function |
|---|---|---|---|
| VPARBFB13_v1_420099 | _ | 304 | 2,3-dihydroxy-p-cumate-3,4-dioxygenase (CmtC) |
| VPARBFB13_v1_1740011 | _ | 276 | 2,3-dihydroxyphenylpropionate 1,2-dioxygenase |
| VPARBFB13_v1_3050062 | *nicX* | 348 | 2,5-dihydroxypyridine 5,6-dioxygenase |
| VPARBFB13_v1_2080056 | _ | 211 | 3-mercaptopropionate dioxygenase |
| VPARBFB13_v1_4840024 | _ | 197 | Cinnamic acid dioxygenase, small subunit |
| VPARBFB13_v1_250002 | _ | 408 | Trans-cinnamate dioxygenase ferredoxin reductase subunit |
| VPARBFB13_v1_1510004 | *ygiD* | 309 | 4,5-DOPA dioxygenase extradiol |
| VPARBFB13_v1_4490055 | *hpd* | 376 | 4-hydroxyphenylpyruvate dioxygenase |
| VPARBFB13_v1_6000006 | _ | 262 | 4-hydroxyphenylpyruvate dioxygenase |
| VPARBFB13_v1_4780024 | _ | 219 | Alkylated DNA repair dioxygenase AlkB |
| VPARBFB13_v1_890004 | *tfdA* | 298 | 2,4-dichlorophenoxyacetate dioxygenase |
| VPARBFB13_v1_6140039 | *tfdA* | 293 | 2,4-dichlorophenoxyacetate dioxygenase |
| VPARBFB13_v1_5800007 | *atsK* | 322 | Sulfate ester dioxygenase |
| VPARBFB13_v1_2120016 | *tauD* | 283 | Alpha-ketoglutarate-dependent taurine dioxygenase |
| VPARBFB13_v1_3050001 | _ | 349 | Alpha-ketoglutarate-dependent taurine dioxygenase |
| VPARBFB13_v1_1010042 | *andAa* | 411 | Anthranilate 1,2-dioxygenase, reductase component |
| VPARBFB13_v1_3050066 | _ | 366 | Anthranilate dioxygenase reductase |

| | | | |
|---|---|---|---|
| VPARBFB13_v1_380052 | _ | 352 | Aromatic ring-hydroxylating dioxygenase subunit alpha |
| VPARBFB13_v1_640013 | _ | 377 | Aromatic ring-hydroxylating dioxygenase subunit alpha |
| VPARBFB13_v1_1740012 | _ | 112 | Aromatic ring-opening dioxygenase subunit LigA |
| VPARBFB13_v1_620005 | _ | 277 | Aromatic ring-opening dioxygenase, catalytic subunit, |
| VPARBFB13_v1_5890034 | _ | 155 | Aromatic-ring-hydroxylating dioxygenase |
| VPARBFB13_v1_420107 | _ | 161 | Aromatic-ring-hydroxylating dioxygenase subunit beta |
| VPARBFB13_v1_420091 | *benB* | 165 | Benzoate 1,2-dioxygenase subunit beta |
| VPARBFB13_v1_170032 | _ | 435 | Benzoate/toluate 1,2-dioxygenase alpha subunit |
| VPARBFB13_v1_170031 | _ | 165 | Benzoate/toluate 1,2-dioxygenase beta subunit |
| VPARBFB13_v1_3300008 | *bphC* | 200 | Biphenyl-2,3-diol 1,2-dioxygenase 3 |
| VPARBFB13_v1_1850032 | _ | 148 | Catechol 2,3-dioxygenase |
| VPARBFB13_v1_4780048 | _ | 134 | Catechol 2,3-dioxygenase |
| VPARBFB13_v1_4840035 | _ | 126 | Catechol 2,3-dioxygenase |
| VPARBFB13_v1_590022 | *linE* | 317 | Chlorohydroquinone/hydroquinone 1,2-dioxygenase |
| VPARBFB13_v1_2990001 | _ | 212 | Cysteine dioxygenase |
| VPARBFB13_v1_590050 | _ | 515 | Dioxygenase |
| VPARBFB13_v1_4780082 | _ | 490 | Dioxygenase |
| VPARBFB13_v1_3410048 | _ | 116 | DOPA 4,5-dioxygenase |
| VPARBFB13_v1_4900006 | _ | 299 | Fe(2+)/alpha-ketoglutarate-dependent dioxygenase LpxO |
| VPARBFB13_v1_2200015 | _ | 359 | Gentisate 1,2-dioxygenase |
| VPARBFB13_v1_2360015 | *hmgA* | 437 | Homogentisate 1,2-dioxygenase |
| VPARBFB13_v1_1820005 | *chqB* | 292 | Hydroxyquinol 1,2-dioxygenase |
| VPARBFB13_v1_2150040 | _ | 177 | Hydroxyquinol 1,2-dioxygenase |
| VPARBFB13_v1_4480016 | *ndoR* | 331 | Napht. 1,2-dioxygenase, reductase component |
| VPARBFB13_v1_3050067 | *ndoA* | 106 | Napht. 1,2-dioxygenase system, ferredoxin component |
| VPARBFB13_v1_4480011 | *nagAb* | 107 | Naphth. 1,2-dioxygenase/salicylate 5-hydroxylase, ferredoxin |
| VPARBFB13_v1_3050064 | _ | 172 | Ortho-halobenzoate 1,2-dioxygenase beta-ISP protein OhbA |
| VPARBFB13_v1_420101 | *cmtAb* | 425 | p-cumate 2,3-dioxygenase system, large oxygenase component |
| VPARBFB13_v1_420100 | *cmtAc* | 162 | p-cumate 2,3-dioxygenase system, small oxygenase component |
| VPARBFB13_v1_1430030 | *pobA* | 410 | Phenoxybenzoate dioxygenase subunit alpha |
| VPARBFB13_v1_40044 | *pobB* | 324 | Phenoxybenzoate dioxygenase subunit beta |
| VPARBFB13_v1_380051 | *pobB* | 323 | Phenoxybenzoate dioxygenase subunit beta |
| VPARBFB13_v1_1430024 | *pobB* | 329 | Phenoxybenzoate dioxygenase subunit beta |
| VPARBFB13_v1_5890025 | _ | 162 | Phenylpropionate dioxygenase |
| VPARBFB13_v1_5890024 | _ | 439 | Phenylpropionate dioxygenase, large terminal subunit |
| VPARBFB13_v1_5800001 | _ | 278 | Phytanoyl-CoA dioxygenase |
| VPARBFB13_v1_1910004 | _ | 181 | Predicted 2-oxoglutarate-and Fe(II)-dependent dioxygenase YbiX |
| VPARBFB13_v1_2150009 | *pcaG* | 205 | Protocatechuate 3,4-dioxygenase alpha chain |
| VPARBFB13_v1_2150010 | *pcaH* | 239 | Protocatechuate 3,4-dioxygenase beta chain |
| VPARBFB13_v1_1010030 | _ | 450 | Phenylpropionate dioxygenase, large terminal subunit |

| | | | |
|---|---|---|---|
| VPARBFB13_v1_1010031 | _ | 179 | Putative 3-phenylpropanoate dioxygenase |
| VPARBFB13_v1_5010018 | _ | 298 | putative 2,4-dichlorophenoxyacetate dioxygenase |
| VPARBFB13_v1_3890057 | _ | 393 | Putative dioxygenase VC_1345 |
| VPARBFB13_v1_570062 | _ | 420 | Putative Hydroxyquinol 1,2-dioxygenase |
| VPARBFB13_v1_170033 | _ | 192 | Putative Phthalate 4,5-dioxygenase |
| VPARBFB13_v1_2830009 | _ | 676 | Putative Phthalate 4,5-dioxygenase |
| VPARBFB13_v1_3720018 | _ | 234 | Quercetin 2,3-dioxygenase |
| VPARBFB13_v1_2070005 | _ | 304 | Taurine dioxygenase |
| VPARBFB13_v1_3740030 | *tauD* | 285 | Taurine dioxygenase, 2-oxoglutarate-dependent |
| VPARBFB13_v1_3740031 | *tauD* | 279 | Taurine dioxygenase, 2-oxoglutarate-dependent |
| VPARBFB13_v1_4350010 | *tphA1II* | 344 | Terephthalate 1,2-dioxygenase, reductase component 2 |
| VPARBFB13_v1_4350007 | *tphA2I* | 418 | Terephthalate 1,2-dioxygenase, subunit alpha 1 |
| VPARBFB13_v1_4350008 | *tphA3I* | 155 | Terephthalate 1,2-dioxygenase, subunit beta 1 |
| VPARBFB13_v1_420092 | *xylX* | 445 | Toluate 1,2-dioxygenase subunit alpha |
| VPARBFB13_v1_4840026 | _ | 455 | Biphenyl-2,3-dioxygenase subunit alpha |
| VPARBFB13_v1_590019 | _ | 293 | Tryptophan 2,3-dioxygenase |
| VPARBFB13_v1_1620042 | *kynA* | 317 | Tryptophan 2,3-dioxygenase |
| VPARBFB13_v1_2900001 | *kynA* | 283 | Tryptophan 2,3-dioxygenase |

**Table S5:** Annotation and predicted function of the proposed BTEX catabolic genes detected in the genome of *V. paradoxus* strain BFB1_13

| **Locus tag (MaGe)** | **Gene** | **Length (aa)*** | **Putative function** | **EC number** |
|---|---|---|---|---|
| VPARBFB13_v1_420070 | *tmoA* | 496 | Toluene-4-monooxygenase, hydroxylase component subunit α | |
| VPARBFB13_v1_420069 | *tmoB* | 90 | Toluene-4-monooxygenase, hydroxylase component subunit γ | |
| VPARBFB13_v1_420068 | *tmoC* | 111 | Toluene-4-monooxygenase, ferredoxin component | 1.14.13.236 |
| VPARBFB13_v1_420067 | *tmoD* | 102 | Toluene-4-monooxygenase, effector component | |
| VPARBFB13_v1_420066 | *tmoE* | 328 | Toluene-4-monooxygenase, hydroxylase component subunit β | |
| VPARBFB13_v1_420065 | *tmoF* | 338 | Toluene-4-monooxygenase, electron transfer component | 1.18.1.3 |
| VPARBFB13_v1_420086 | *dmpK* | 72 | Phenol hydroxylase P0 protein | |
| VPARBFB13_v1_60032 | | 92 | | |
| VPARBFB13_v1_420085 | *dmpL* | 330 | Phenol hydroxylase P1 protein | |
| VPARBFB13_v1_60031 | | 305 | | |
| VPARBFB13_v1_420084 | | 89 | | |
| VPARBFB13_v1_420123 | *dmpM* | 89 | Phenol hydroxylase P2 protein | |
| VPARBFB13_v1_60029 | | 98 | | |
| VPARBFB13_v1_420083 | | 519 | | |
| VPARBFB13_v1_420122 | *dmpN* | 519 | Phenol hydroxylase P3 protein | 1.14.13.7 |
| VPARBFB13_v1_60027 | | 531 | | |
| VPARBFB13_v1_420082 | | 118 | | |
| VPARBFB13_v1_420121 | *dmpO* | 118 | Phenol hydroxylase P4 protein | |
| VPARBFB13_v1_60026 | | 120 | | |
| VPARBFB13_v1_420081 | | 354 | | |
| VPARBFB13_v1_420120 | *dmpP* | 354 | Phenol hydroxylase P5 protein | |
| VPARBFB13_v1_60025 | | 352 | | |
| VPARBFB13_v1_60023 | | 310 | | |
| VPARBFB13_v1_420080 | | 314 | | |
| VPARBFB13_v1_420119 | *xylE* | 314 | Catechol 2,3-dioxygenase | 1.13.11.2 |
| VPARBFB13_v1_1010044 | | 314 | | |
| VPARBFB13_v1_3270059 | | 314 | | |

| | | | | |
|---|---|---|---|---|
| VPARBFB13_v1_60022<br>VPARBFB13_v1_420078<br>VPARBFB13_v1_420117<br>VPARBFB13_v1_1010045 | *dmpC* | 484<br>484<br>484<br>484 | 2-hydroxymuconate-6-semialdehyde dehydrogenase | 1.2.1.85 |
| VPARBFB13_v1_60016<br>VPARBFB13_v1_420071<br>VPARBFB13_v1_1010050 | *praC, xylH* | 63<br>63<br>63 | 2-hydroxy-muconate tautomerase | 5.3.2.6 |
| VPARBFB13_v1_60021<br>VPARBFB13_v1_1010051 | *dmpD* | 274<br>276 | 2-hydroxy-muconate semialdehyde hydrolase | 3.7.1.9 |
| VPARBFB13_v1_3270057 | *todF* | 280 | 2-hydroxy-6-oxohepta-2,4-dienoate hydrolase | 3.7.1.25 |
| VPARBFB13_v1_60017<br>VPARBFB13_v1_420072<br>VPARBFB13_v1_420112<br>VPARBFB13_v1_1010049 | *dmpH, xylI* | 262<br>262<br>262<br>263 | 4-oxalocrotonate decarboxylase | 4.1.1.77 |
| VPARBFB13_v1_60020<br>VPARBFB13_v1_420077<br>VPARBFB13_v1_420116<br>VPARBFB13_v1_1010046 | *mhpD, xylJ* | 260<br>264<br>264<br>262 | 2-keto-4-pentenoate/2-hydroxyhexa-2,4-dienoate hydratase | 4.2.1.80/4.2.1.132 |
| VPARBFB13_v1_60018<br>VPARBFB13_v1_420073<br>VPARBFB13_v1_420113<br>VPARBFB13_v1_1010048 | *bphI, xylK* | 358<br>347<br>347<br>342 | 4-hydroxy-2-oxovalerate/4-hydroxy-2-oxohexanoate aldolase | 4.1.3.39/4.1.3.43 |
| VPARBFB13_v1_420063 | *xylM* | 211 | Xylene monooxygenase | 1.14.15.26 |
| VPARBFB13_v1_4840055 | *xylB* | 367 | Aryl-alcohol dehydrogenase | 1.1.1.90 |
| VPARBFB13_v1_4890002 | *xylC* | 400 | Salicylaldehyde /vanillin/benzaldehyde dehydrogenase | 1.2.1.7/1.2.1.28 |
| VPARBFB13_v1_170032<br>VPARBFB13_v1_420092 | *benA-xylX* | 434<br>444 | Benzoate/toluate 1,2-dioxygenase α subunit | 1.14.12.10/1.14.12.- |
| VPARBFB13_v1_170031<br>VPARBFB13_v1_420091 | *benB-xylY* | 164<br>164 | Benzoate/toluate 1,2-dioxygenase β subunit | |
| VPARBFB13_v1_420090 | *benD-xylL* | 273 | Dihydroxy cyclohexadiene carboxylate dehydrogenase | 1.3.1.25 |
| VPARBFB13_v1_1010030 | *ndoB* | 449 | Naphthalene 1,2-dioxygenase system, large oxygenase component | 1.14.12.12 |

| | | | | |
|---|---|---|---|---|
| VPARBFB13_v1_3050067<br>VPARBFB13_v1_4480011 | *ndoA*<br>*nagAb* | 105<br>106 | Naphthalene 1,2-dioxygenase/ferredoxin component | 1.18.1.7 |
| VPARBFB13_v1_4480016 | *nagAa, ndoR* | 330 | Naphthalene 1,2-dioxygenase, reductase component | 1.18.1.7 |
| VPARBFB13_v1_1730008 | *styA* | 424 | Styrene monooxygenase | 1.14.14.11 |
| VPARBFB13_v1_600002<br>VPARBFB13_v1_3890017 | *styD* | 443<br>476 | Phenylacetaldehyde dehydrogenase | 1.2.1.39 |
| VPARBFB13_v1_2360015 | *hmgA* | 436 | Homogentisate 1,2-dioxygenase | 1.13.11.5 |
| VPARBFB13_v1_2140079<br>VPARBFB13_v1_5970002 | *maiA* | 222<br>224 | Maleyilacetoacetate isomerase | 5.2.1.2 |
| VPARBFB13_v1_1430037<br>VPARBFB13_v1_2360013 | *fah* | 448<br>422 | Fumarylacetoacetase | 3.7.1.2 |

*-aa, amino acids

**Table S6:** The number and closest affiliation of toluene 4-monnooxygenase, phenol-2-hydroxylase and catechol 2,3-dioxygenase encoding genes detected in the genome of *V. paradoxus* strain BFB1_13

| **Locus Tag (MaGe)** | **Gene** | **Best match according to the UniProt BLAST search / Amino acid sequence similarities are shown (%)** | | | |
|---|---|---|---|---|---|
| | | UniProtKB/TrEMBL (Unreviewed) | | UniProtKB/Swiss-Prot (Reviewed) | |
| VPARBFB13_v1_420070 | *tmoA* | *Methylibium petroleiphilum* LMG 22953 | 86 | *Pseudomonas mendocina* ATCC 25411 | 64 |
| VPARBFB13_v1_420069 | *tmoB* | *Rugosibacter aromaticivorans* Ca6 | 79 | *Pseudomonas mendocina* ATCC 25411 | 49 |
| VPARBFB13_v1_420068 | *tmoC* | *Thiomonas delicata* DSM 5494 | 81 | *Pseudomonas mendocina* ATCC 25411 | 49 |
| VPARBFB13_v1_420067 | *tmoD* | *Methylibium petroleiphilum* LMG 22953 | 70 | *Pseudomonas mendocina* ATCC 25411 | 52 |
| VPARBFB13_v1_420066 | *tmoE* | *Methylibium petroleiphilum* LMG 22953 | 84 | *Pseudomonas mendocina* ATCC 25411 | 61 |
| VPARBFB13_v1_420065 | *tmoF* | *Pseudoxanthomonas spadix* Bda-59 | 66 | *Pseudomonas resinovorans* ATCC 14235 | 44 |
| VPARBFB13_v1_420086 | *dmpK* | *Burkholderiales bacterium* PBB5 | 65 | *Pseudomonas* sp. strain CF600 | 44 |
| VPARBFB13_v1_60032 | | *Variovorax* sp. PDC80 | 100 | *Pseudomonas* sp. strain CF600 | 46 |
| VPARBFB13_v1_420085 | *dmpL* | *Hydrogenophaga* sp. T4 | 75 | *Pseudomonas* sp. strain CF600 | 48 |
| VPARBFB13_v1_60031 | | *Variovorax* sp. PDC80 | 99 | *Pseudomonas* sp. strain CF600 | 53 |
| VPARBFB13_v1_420084 | *dmpM* | *Hydrogenophaga* sp. T4 | 79 | *Pseudomonas* sp. strain CF600 | 42 |
| VPARBFB13_v1_420123 | | *Hydrogenophaga* sp. T4 | 79 | *Pseudomonas* sp. strain CF600 | 42 |
| VPARBFB13_v1_60029 | | *Variovorax* sp. PDC80 | 99 | *Pseudomonas sp*. strain *CF600* | 50 |
| VPARBFB13_v1_420083 | *dmpN* | *Leptothrix cholodnii* ATCC 51168 | 88 | *Pseudomonas* sp. strain CF600 | 66 |
| VPARBFB13_v1_420122 | | *Leptothrix cholodnii* ATCC 51168 | 88 | *Pseudomonas* sp. strain CF600 | 66 |
| VPARBFB13_v1_60027 | | *Variovorax* sp. PDC80 | 100 | *Pseudomonas* sp. strain CF600 | 66 |
| VPARBFB13_v1_420082 | *dmpO* | *Hydrogenophaga* sp. T4 | 87 | *Pseudomonas* sp. strain CF600 | 44 |
| VPARBFB13_v1_420121 | | *Hydrogenophaga* sp. T4 | 87 | *Pseudomonas* sp. strain CF600 | 44 |
| VPARBFB13_v1_60026 | | *Variovorax* sp. PDC80 | 100 | *Pseudomonas* sp. strain CF600 | 47 |
| VPARBFB13_v1_420081 | *dmpP* | *Pseudoxanthomonas spadix* BDa-59 | 81 | *Pseudomonas* sp. strain CF600 | 58 |
| VPARBFB13_v1_420120 | | *Pseudoxanthomonas spadix* BDa-59 | 81 | *Pseudomonas* sp. strain CF600 | 58 |
| VPARBFB13_v1_60025 | | *Variovorax* sp. PDC80 | 98 | *Pseudomonas* sp. strain CF600 | 58 |
| VPARBFB13_v1_3270059 | *xylE* | *Caenimonas* sp. HX-9-20 | 92 | *Pseudomonas putida* DSM 291 | 44 |
| VPARBFB13_v1_1010044 | | *Noviherbaspirillum* sp. Root189 | 86 | *Pseudomonas putida* DSM 291 | 44 |
| VPARBFB13_v1_420080 | | *Caenimonas* sp. HX-9-20 | 92 | *Pseudomonas putida* DSM 291 | 43 |
| VPARBFB13_v1_420119 | | *Caenimonas* sp. HX-9-20 | 92 | *Pseudomonas putida* DSM 291 | 43 |
| VPARBFB13_v1_60023 | | *Variovorax* sp. PDC80 | 100 | *Pseudomonas putida* DSM 291 | 62 |
